# Contrasting Effects of Wild *Arachis* Dehydrin Under Abiotic and Biotic Stresses

**DOI:** 10.3389/fpls.2019.00497

**Published:** 2019-04-18

**Authors:** Ana Paula Zotta Mota, Thais Nicolini Oliveira, Christina Cleo Vinson, Thomas Christopher Rhys Williams, Marcos Mota do Carmo Costa, Ana Claudia Guerra Araujo, Etienne G. J. Danchin, Maria Fatima Grossi-de-Sá, Patricia Messenberg Guimaraes, Ana Cristina Miranda Brasileiro

**Affiliations:** ^1^EMBRAPA Recursos Genéticos e Biotecnologia, Brasília, Brazil; ^2^Departamento de Biologia Celular e Molecular, Universidade Federal do Rio Grande do Sul, Porto Alegre, Brazil; ^3^Departamento de Botânica, Universidade de Brasília, Brasília, Brazil; ^4^INRA, Université Côte d’Azur, CNRS, ISA, Sophia-Antipolis, France

**Keywords:** *Arabidopsis*, drought, freezing, genome-wide, *Meloidogyne*, root-knot nematode

## Abstract

Plant dehydrins (DNHs) belong to the LEA (Late Embryogenesis Abundant) protein family and are involved in responses to multiple abiotic stresses. DHNs are classified into five subclasses according to the organization of three conserved motifs (K-; Y-; and S-segments). In the present study, the DHN protein family was characterized by molecular phylogeny, exon/intron organization, protein structure, and tissue-specificity expression in eight Fabaceae species. We identified 20 DHN genes, encompassing three (Y_n_SK_n_, SK_n_, and K_n_) subclasses sharing similar gene organization and protein structure. Two additional low conserved DHN Φ-segments specific to the legume SK_n_-type of proteins were also found. The *in silico* expression patterns of DHN genes in four legume species (*Arachis duranensis, A. ipaënsis, Glycine max*, and *Medicago truncatula*) revealed that their tissue-specific regulation is associated with the presence or absence of the Y-segment. Indeed, DHN genes containing a Y-segment are mainly expressed in seeds, whereas those without the Y-segment are ubiquitously expressed. Further qRT-PCR analysis revealed that, amongst stress responsive dehydrins, a SK_n_-type DHN gene from *A. duranensis* (*AdDHN1*) showed opposite response to biotic and abiotic stress with a positive regulation under water deficit and negative regulation upon nematode infection. Furthermore, transgenic *Arabidopsis* lines overexpressing (OE) *AdDHN1* displayed improved tolerance to multiple abiotic stresses (freezing and drought) but increased susceptibility to the biotrophic root-knot nematode (RKN) *Meloidogyne incognita*. This contradictory role of *AdDHN1* in responses to abiotic and biotic stresses was further investigated by qRT-PCR analysis of transgenic plants using a set of stress-responsive genes involved in the abscisic acid (ABA) and jasmonic acid (JA) signaling pathways and suggested an involvement of DHN overexpression in these stress-signaling pathways.

## Introduction

The dehydrin (DHN) family of proteins, or dehydration proteins, belongs to group II of the LEA (Late Embryogenesis Abundant) proteins and is considered the most important LEA group due to its involvement in tolerance to several abiotic stresses. DHNs are defined by the presence of three conserved motifs, named the K-, Y-, and S-segments, that classify DHNs into five subclasses according to their number and organization: Y_n_SK_n_; K_n_; SK_n_; K_n_S; and Y_n_K_n_ (Close, 1996). DHN proteins typically contain at least one copy of the signature K-segment, a 15-amino acid lysine-rich repeat (EKKGIMDKIKEKLPG) near the C-terminus (Malik et al., 2017). The two other conserved motifs, despite being common in DHN, are not essential to characterize the protein: the Y-segment ([V/T]D[E/Q]YGNP), near to the N-terminus, and the S-segment, a group of four to eight serine residues (Malik et al., 2017). The K-, Y-, and S-segments are separated by poorly conserved motifs, called Φ-segments, that are usually rich in glycine and polar amino acids (Graether and Boddington, 2014).

DHNs often accumulate in vegetative tissues of higher plants in response to different types of abiotic stresses, such as low temperature, drought, salinity, or wounding, and this response represents part of the molecular arsenal developed by plants to withstand the damaging effects of the intracellular water loss caused by these stresses (Graether and Boddington, 2014). However, despite considerable research, the mechanisms underlying DHN responses to dehydration remain unclear and each type of DHN might have a specific function. It is generally accepted that DHNs act as chaperones to prevent the aggregation, damage, and inactivation of proteins during stress imposition (Hara et al., 2005; Kovacs et al., 2008). The highly disordered nature of DHN structure also contributes to biochemical properties that permit these proteins to function in enzyme activity protection, membrane binding and stabilization, ROS scavenging, and ion sequestration (Banerjee and Roychoudhury, 2016).

Plant DHN gene expression is generally modulated in response to abiotic stress and varies according to the type of condition imposed and the subclass to which they belong (Abedini et al., 2017). In particular, the DHN SK_n_-types are commonly associated with cold tolerance and found near the plasma membrane where they exert a protective role (Graether and Boddington, 2014). In addition to cold stress, there is evidence that DHNs belonging to the SK_n_-type can also enhance the ability of plants to cope with multiple and simultaneous abiotic stresses. For example, the overexpression in tobacco plants of SK_n_-type genes isolated from freezing-tolerant species (arctic chickweed and Chinese mei) improved tolerance to cold, drought, salt, and osmotic stress (Hill et al., 2016; Bao et al., 2017). Also, a SK_3_ DHN from wild tomato and a SK_2_ from rubber-tree enhanced tolerance to more than one abiotic stress in transgenic tomato and *Arabidopsis* plants, respectively (Liu H. et al., 2015; Cao et al., 2017). Likewise, the overexpression of a SK_3_ DHN from a stress-tolerant *Musa* genotype led to superior performance in a commercial cultivar of transgenic banana under both drought and salinity (Shekhawat et al., 2011).

In contrast, some SK_n_-type genes appear to be stress-specific. For instance, those isolated from cold-adapted species (wheat and wild potato), that have been shown to enhance tolerance to freezing in transgenic strawberry and cucumber, respectively (Houde et al., 2004; Yin et al., 2006); or that isolated from a drought-tolerant wild olive species, which increased tolerance to water deficit in transgenic *Arabidopsis* (Chiappetta et al., 2015). Similarly, a sorghum SK_3_ increased protection against oxidative stress when overexpressed in tobacco (Halder et al., 2018), and a SK_3_ from the halophyte *Ipomoea pes-caprae* increased the salt tolerance when overexpressed in *Arabidopsis* (Zhang et al., 2018).

Unlike in abiotic stress, the involvement of plant DHNs in biotic stress is poorly studied, with few studies showing modulation of DHN expression associated with responses to fungal infection either on its own or in association with abiotic stress (Turco et al., 2004; Yang et al., 2012). Although DHNs are known to respond to wounding and exogenous hormones that play a vital role in pathogen defense signaling and disease resistance, such as Abscisic acid (ABA), Jasmonic acid (JA), Salicylic acid (SA), and Ethylene (ET), their role in plant defense against pathogens remains to be elucidated (Shen et al., 2004; Hanin et al., 2011; Rosales et al., 2014). In addition, despite the importance of DHNs in abiotic stress tolerance mechanisms, little is known about their potential role in legume crop yields in stress-prone environments (Rémus-Borel et al., 2010; Araújo et al., 2015). Furthermore, the evolutionary history of the legume DHN family has never been phylogenetically investigated, with a previous review focusing only on overall sequence similarity analysis of LEA proteins in six legume species (Battaglia et al., 2013).

To date, there is only one report on the use of DHN genes isolated from legume species for the improvement of stress tolerance in crop legumes through transgenic approaches (Xie et al., 2012). However, rapid advances and the improvement in accuracy of bioinformatics approaches as well as re-annotated versions of legume genomes, including the recent genome sequencing of two *Arachis* species (Bertioli et al., 2016), can contribute to new insights into the molecular function and evolution of the DHN family in legumes.

In the present study, we characterized the DHN gene family in legumes regarding their phylogenetic classification, chromosomal localization, duplication events, molecular structure and spatial expression patterns. We then focused on the transcriptional behavior of a DHN gene from the wild peanut *Arachis duranensis* (*AdDHN1*) in response to abiotic and biotic stresses, shedding light on its role in stress responses. The overexpression of *AdDHN1* in *Arabidopsis* plants improved their tolerance to multiple abiotic stresses (freezing and drought) but increased their susceptibility to the biotrophic root-knot nematode (RKN) *Meloidogyne incognita*. This trade-off effect: improved resistances to abiotic stresses vs. increased susceptibility to biotic stress is an important finding that must be taken into consideration in research into the improvement of crop plants.

## Materials and Methods

### Identification and Phylogenetic Analysis of Dehydrins in Fabaceae

The complete genomes of eight species (*Arachis duranensis, A. ipaënsis, Cicer arietinum, Cajanus cajan, Glycine max, Lotus japonicus, Medicago truncatula*, and *Phaseolus vulgaris*) belonging to the Fabaceae family were downloaded from their respective public databases (Supplementary Table 1). To identify the putative DHN proteins in these eight proteomes, we used two independent approaches. First, the PFAM (Finn et al., 2016) dehydrin domain (PF00257) was used as a query for the program hmmsearch, from the HMMER3 suite (Mistry et al., 2013) against the predicted proteome of each species. In the second approach, the K-segment described by Malik et al. (2017) was used as input for the Find Individual Motif Occurrences (FIMO) software (Grant et al., 2011), applying a threshold *q*-value of < 0.05. The proteins found using both approaches were verified for their motifs, presence and organization. Only proteins with more than half of the total length of the PF00257 domain (168 aa) and with a canonical dehydrin motif organization were considered in the present study.

All the identified putative DHN protein sequences were aligned using the MAFFT software with automatic detection of the most appropriate alignment strategy (Katoh et al., 2002). The poorly aligned regions (more than 10% of gaps) were eliminated with the trimAl software (Capella-Gutiérrez et al., 2009). We performed the phylogenetic analysis of the selected protein sequences using RAxML software (Stamatakis, 2006), with automatic search of the fittest evolutionary model and parameters and a bootstrap search automatically stopped upon congruence.

### Synteny Analysis, Duplication Pattern, and Gene/Protein Structure of Dehydrins in Fabaceae

We retrieved the physical location of the putative DHN genes in the chromosomes of the eight Fabaceae species from the GFF-formatted file in their respective databases. The MCScanX software (Wang et al., 2012) was used to determine the syntenic relationship and duplication patterns between these species and Circa^1^ to plot graphical representation of DHN genes location and their syntenic relationships.

The intron/exon organization of dehydrin genes was extracted from the GFF-formatted file of each genome and submitted to the GSDS software^2^ for graphical representation. We predicted the consensus sequence of the DHN motifs of the proteins using the Multiple Expectation maximization for Motif Elicitation (MEME) (Bailey et al., 2009).

### Spatial Expression Pattern of Fabaceae Dehydrin Genes

The gene expression atlas of *A. duranensis, A. ipaënsis, G. max*, and *M. truncatula*^3^ was used to analyze the spatial expression patterns of DHNs. Respective expression values, represented as FPKM, were retrieved in table format and the mean FPKM values for each of the following tissues were determined: dry seeds, roots, leaves and stems in each species. In addition, the RT-PCR analysis of transcript abundance of *G. max* DHN genes by Yamasaki et al. (2013) was also considered in our analysis.

### Analysis of Dehydrin Expression in Wild *Arachis* in Response to Stress

Our previously published transcriptome RNA-Seq data were exploited to determine the *in silico* expression profile of the two *A. duranensis* DHN genes in response to both abiotic (water deficit) and biotic (nematode inoculation) stresses (Mota et al., 2018; Vinson et al., 2018). Quantitative RT-PCR (qRT-PCR) analysis was also conducted, essentially as described by Morgante et al. (2013), to determine the relative expression of the *AdDHN1* and *AdDHN2* genes (Supplementary Table 2) in *A. duranensis* plants under dehydration and upon nematode infection, using the RNA isolated in Vinson et al. (2018) and Mota et al. (2018), respectively. ACT1 and UBI2 were used as reference genes for *Arachis* root samples subjected to dehydration; and 60S and GAPDH for samples inoculated with nematodes, in accordance with Morgante et al. (2011).

### *AdDHN1* Cloning

To identify the complete coding sequence of *AdDHN1*, the Aradu.IF4XP gene model^4^ was aligned with the four best BLASTn hits of *A. duranensis* databases available on NCBI^5^. The consensus sequence (675 bp) was synthesized and cloned (Epoch Life Science Inc., TX, United States), under the control of the *Arabidopsis* actin 2 promoter (ACT-2) and the nopaline synthase (NOS) terminator, at the XhoI restriction site of pPZP-201BK-EGFP (Chu et al., 2014). This binary vector, hereafter called pPZP-AdDHN1, also contains two additional cassettes for the constitutive expression of the enhanced green fluorescent protein (eGFP) reporter gene and the hygromycin phosphotransferase (hpt) selection marker gene. The pPZP-AdDHN1 binary vector was then introduced into the disarmed *Agrobacterium tumefaciens* strain “GV3101” by standard electroporation protocol. Transformed colonies were selected by PCR using primer pairs flanking the eGFP or *AdDHN1* sequences (Supplementary Table 2).

### *Arabidopsis* Transformation

Wild type (WT) *Arabidopsis thaliana* ecotype Columbia (Col-0) was transformed with *A. tumefaciens* containing the pPZP-AdDHN1 vector using the floral dip method (Clough and Bent, 1998) and maintained in a growth chamber at 21°C, 60% relative humidity, 12 h photoperiod. Seeds originating from the dipped-plants were germinated on plates containing solid (0.8% w/v agar) half-strength Murashige and Skoog (MS) basal medium (Sigma-Aldrich, St. Louis City, United States) with sucrose (30 g/L) and hygromycin (15 mg/L). Hygromycin-resistant T0 plants were transferred to pots containing substrate (Carolina Soil^^®^^, CSC, Brazil) and maintained in the controlled growth chamber to produce the T1 generation. Transformants were then screened repeatedly for hygromycin resistance and grown on to obtain homozygous T3 generation *AdDHN1* overexpressing (OE) lines for further analysis.

### Stress Assays in Transgenic *Arabidopsis* Overexpressing (OE) *AdDHN1*

Seeds harvested from 13 OE lines at T3 generation were used to determine the effect of freeze-shock treatment on seedling growth. Two weeks old seedlings germinated on MS medium plates (12 individuals per OE line and WT per plate, three plates per line) were placed in a temperature-regulated freezer at -18°C for 1 h in the dark. After complete freezing of the MS medium, the seedlings on plates were returned to normal controlled growth conditions. The survival rate after the freezing treatment was assessed by recording the number of seedlings that regained turgidity and displayed a normal phenotype compared to that of non-treated seedlings, 3 days after the treatment. Seedlings that showed healthy growth in treated and non-treated plates were further used for quantification of total soluble sugars (Buysse and Merckx, 1993) and qRT-PCR analysis (see below).

The *Arabidopsis* OE lines with enhanced freezing tolerance were then selected for subsequent stress assays. For that, OE and WT plants, grown in the controlled growth chamber, were submitted to a gradual water deficit (dry-down) treatment and to infection with *M. incognita.* We analyzed the data using single factor ANOVA (*P* < 0.05).

For the dry-down assay, irrigation of 3 weeks old plants grown on substrate was interrupted for a group of 10 individuals per OE line and the WT (stressed group) during 8 days whilst the control group of individuals was kept under irrigated conditions, i.e., around 70% of field capacity (FC). During the assay, two SPAD chlorophyll meter readings (SCMR; SPAD-502, Konica Minolta Sensing, Japan) was recorded every 2 days from the same leaf of each individual. At the end of the water stress (8th day), we collected three leaf discs (0.4 cm^2^) per individual to assess Relative Water Content (RWC) according to de Brito et al. (2011). The aerial and root plant fresh biomass were also determined at the end of the assay and the leaf area was estimated using the Rosette Tracker software (De Vylder et al., 2012).

For the *M. incognita* bioassay, roots from 4 weeks old plants grown on a 2:1 sand:substrate mixture (v:v) were inoculated with approximately 500 J2 infective larvae of *M. incognita*, essentially as described by Morgante et al. (2013). At 60 days after inoculation (DAI), roots were stained with acid fuchsin and the number of nematode females on roots (10 individuals per OE line and WT) assessed under a stereomicroscope.

### Analysis of Transcription Levels in *Arabidopsis* by qRT-PCR

We conducted qRT-PCR analysis in the T3 seedlings to study the expression levels of the *AdDHN1* transgene and other stress-responsive genes in *Arabidopsis* OE lines and WT plants. Total RNA was extracted using an RNeasy Plant Mini Kit (Qiagen, Hilden, Germany), treated with DNAse and reverse transcribed as previously described (Morgante et al., 2013). qRT-PCR reactions were performed in three biological replicates on the StepOne Plus Real-Time PCR System (Applied Biosystems, Foster City, United States), as previously described (Vinson et al., 2018), using specific primers (Supplementary Table 2). The online real-time PCR Miner tool (Zhao and Fernald, 2005) was used to estimate the average cycle threshold (Cq) values. The relative quantification (RQ) of mRNA levels was normalized with AtACT2 and AtEF-1α reference genes (Supplementary Table 2) and determined for the *AdDHN1* transgene using the qGENE software^6^ and for the stress-responsive *Arabidopsis* genes using the REST 2009 v. 2.0.13 software (Pfaffl et al., 2002).

## Results

### Identification and Characterization of DHN Genes in Fabaceae

We searched for DHN proteins in the proteomes predicted from the whole genome sequences of eight Fabaceae species belonging to the Papilionoideae sub-family based on the presence of the conserved dehydrin PFAM domain PF00257. Initially, we identified 21 putative DHN proteins in the eight species (Supplementary Table 1). However, two protein sequences from *C. arietinum*, and their corresponding genes, were 100% identical and hence eliminated, yielding a total of 20 non-redundant putative DHN proteins. Based on their motif numbers and organization, we could classify all 20 putative DHNs identified into only three out of the five known subclasses: Y_n_SK_n_, Y_n_K_n_, and SK_n_. Subsequently, we named each DHN according to the corresponding chromosomal position of the gene in each species (Supplementary Table 1).

### Phylogenetic Analyses, Gene Structure, and Protein Motifs of DHNs in Fabaceae Species

We performed a Maximium-likehood (ML) phylogenetic analysis using the 20 deduced DHN protein sequences identified in the eight Fabaceae species. The phylogenetic analysis found the JTT model as the most appropriate and the analysis converged after 599 bootstrap replicates. This analysis could separate, with high confidence values, the putative DHN proteins into two distinct groups, the proteins with a Y-segment (belonging to the Y_n_SK_n_ and Y_n_K_n_ subclasses) and the proteins without a Y-segment (belonging to the SK_n_ subclass) (Supplementary Figure 1). The number of DHN proteins in the two groups was identical (ten in each group). With the exception of *C. cajan*, all the Fabaceae species possessed at least one SK_n_ and one Y_n_SK_n_ DHN protein. *L. japonicus* was the only species with a predicted additional Y_n_K_n_-type DHN. This *L. japonicus* protein holds an outgroup position in the Y-segment DHN clade (Supplementary Figure 1).

**FIGURE 1:**
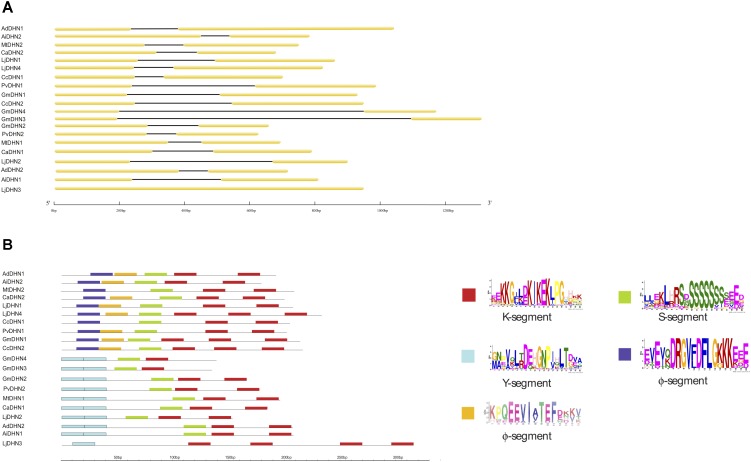
Gene and protein organization of the 20 DHNs in eight Fabaceae species. **(A)** Exon/intron organization of the DHN genes; **(B)** Motif organization and conserved segments of the DHN proteins represented by different colors (left) and their corresponding motif logos (right). The scale length is represented in base pairs (bp) and amino acids.

We further analyzed the structural diversity of the 20 DHN genes through their exon/intron organization. Regardless of their phylogenetically-determined protein group, the legume DHN genes showed a very conserved 2-exon/1-intron organization, except for the *LjDHN3* gene from *L. japonicus* which was devoid of introns and was the only legume DHN gene that did not contain an S-segment (Figure 1A). Notably, the presence of a conserved intron in DHN genes seems to be associated with the presence of an S-segment in *Arabidopsis*, potato and rapeseed (Jiménez-Bremont et al., 2013; Charfeddine et al., 2015; Liang et al., 2016). The protein structures of all 20 legume DHNs showed that, when present, the Y-segment generally occurred in two consecutive copies and the K-segment in one to four copies (Figure 1B and Supplementary Figure 2). In accordance with the phylogenetic analysis (Supplementary Figure 1), half of these proteins presented one or two Y-segments and are therefore classified as Y_n_SK_n_- and Y_n_K_n_-types. All DHNs identified in the eight legumes species showed a single S-segment, except LjDHN3 which lacked this segment (Figure 1B and Supplementary Figure 2). In this study, in addition to the location of the three conserved DHN motifs, we could identify two additional consensus sequences preceding the S-segment, represented by (DRGV[FL]DFLG) and (EE[VA]I[AV]TEF), near the N-terminal region of proteins belonging to the SK_n_-type (Figure 1B and Supplementary Figure 2). These two DHN motifs have not yet been described, and hereafter are considered as Φ-segments, previously noticed as poorly conserved segments located between the K-, S-, and Y-segments (Graether and Boddington, 2014).

We studied the predicted subcellular localization of the 20 legume DHNs using the WoLF PSORT Prediction Software^7^. Interestingly, 19 out of 20 proteins studied were predicted to have a nuclear localization with only the Y_n_SK_n_-type CaDHN1 predicted to be cytoplasmic (Supplementary Table 1). This preferential nuclear localization of legume DHNs is not in accordance with previous studies showing that plant DHNs subcellular localization does not tend to be more nuclear than the cytoplasmic and are present in different parts of the cell (Abedini et al., 2017; Yu et al., 2018). Since the presence of phosphorylation sites is commonly associated with the subcellular localization of DHN proteins in the plant cell (Abedini et al., 2017), a further analysis revealed the presence of one phosphorylation motif and one nuclear localization site (NLS) in legume DHNs. The phosphorylation site (LXRXXS) was identified in 14 out of the 20 DHNs (Supplementary Figure 2 and Supplementary Table 1), regardless of their subclasses, whilst the remaining six DHN proteins that did not present this motif belonged to the Y_n_SK_n_-type (AdDHN2, AiDHN1, GmDHN3, and GmDHN4), SK_n_-type (LjDHN4) and the only example in legumes of the Y_n_K_n_-type (LjDHN3). Despite the absence of a phosphorylation motif, these proteins are still predicted to be in the nucleus. Interestingly, the presence of a NLS was observed only in DHN proteins belonging to the Y_n_SK_n_ subclass, with this specificity also observed in grapevine and barley (Yang et al., 2012; Abedini et al., 2017). The presence of these phosphorylation and NLS sites allow the phosphorylation of the S-segment and has previously been associated with the translocation of DHNs from the cytoplasm to the nucleus (Yang et al., 2012; Abedini et al., 2017; Yu et al., 2018). However, in legumes DHN traffic in the cell could also be associated with other motifs, as LjDHN3, which lacks both phosphorylation motifs, is predicted to be located in the nucleus, whereas CaDHN1, a Y_n_SK_n_-type which contains both phosphorylation motifs, is predicted to have a cytoplasmic location.

### Chromosomal Location and Syntenic Relationships of DHN Genes in Fabaceae Species

The chromosomal location of the DHN genes varied according to the legume species and the number of representatives per species. Interestingly, with the exception of *C. cajan*, which does not possess a Y_n_SK_n_-type DHN, all the species possessed one SK_n_-type and one Y_n_SK_n_-type in physical proximity on the same chromosome (Supplementary Figure 3). The two *C. cajan* DHN genes belonging to the SK_n_ type were distributed on distinct chromosomes (Cc06 and Cc07). *G. max* and *L. japonicus* possess more DHN genes (four representatives each) and, in addition to the co-localization of the conserved SK_n_-type and Y_n_SK_n_-type on a same chromosome (Gm04 for *G. max* and Lj01 for *L. japonicus*) (Supplementary Figure 3), the two extra representatives were on different chromosomes. For *G. max*, the two extra Y_n_SK_n_-type copies were present on chromosomes Gm12 and Gm13. For *L. japonicus*, the extra SK_n_-type DHN was on chromosome Lj05 while the Y_n_K_n_-type, so far specific to this species, was on chromosome Lj02.

From a McScanX analysis at the whole genome level for the eight species, we further focused on the duplication and syntenic relationships of the 20 legume DHN genes (Supplementary Figure 3). The association of one Y_n_SK_n_- and one SK_n_-type representative located on the same chromosome, observed in seven out of eight species (the exception is *C. cajan*), was part of a larger conserved synteny block for six species (*A. duranensis, A. ipaënsis, G. max, L. japonicus, M. truncatula*, and *P. vulgaris*). Surprisingly, the two *C. arietinum* representative genes, although also on the same chromosome, did not show conserved synteny with the conserved blocks from the other six species. Likewise, the extra representatives for *G. max* and *L. japonicus*, distributed on different chromosomes, did not exhibit synteny among the legume species (Supplementary Figure 3).

Previous reports described that the DHN gene family had undergone duplication events in plants, resulting mostly from whole-genome (WGD) and tandem duplications (Yang et al., 2012; Liang et al., 2016). However, this does not seem to be the case for the DHN genes from Fabaceae species, where only one pair of DHN genes from *G. max* (*GmDHN3/GmDHN4*) was considered as duplicated through a WGD/tandem event. These results indicate that except for *G. max*, duplication events of the DHN gene families in Fabaceae probably occurred before speciation, which is also consistent with our phylogenetic analysis (Supplementary Figure 1).

### DHN Gene Spatial Expression in Fabaceae

We analyzed the possible relationship between the presence/absence and organization of the DHN conserved segments and their spatial expression pattern in plant tissues using the expression atlas^8^ publicly available for four (*A. duranensis, A. ipaënsis, G. max*, and *M. truncatula*) of the Fabaceae species and a transcript abundance analysis of *G. max* (Yamasaki et al., 2013). Within a DHN type, the expression across the different species tended to be similar (Supplementary Table 3). Three of the SK_n_-type genes (*AdDHN1, AiDHN2*, and *GmDHN1*) were predicted to be ubiquitous with high FPKM expression values (from 4.26 to 449.51) in dry seeds, roots, leaves, and stems. This expression behavior was confirmed for the *G. max* gene (*GmDHN1*) by Yamasaki et al. (2013) through RT-PCR analysis of seeds, leaves, and stems. The expression of the *MtDHN2* gene from *M. truncatula* was not detected in any of the experiments of Benedito et al. (2008) included in the expression atlas (Supplementary Table 3). Contrastingly, half of the legume DHN genes that belong to the Y_n_SK_n_ type (*AdDHN2, AiDHN1*, and *MtDHN1*) exhibited a more tissue-specific expression, restricted mainly to seeds (FPKM > 16,000 for *M. truncatula*), and a very low gene expression in other tissues. Moreover, expression of the *G. max* Y_n_SK_n_ genes (*GmDHN2* and *GmDHN2*) was only detected in seeds by Yamasaki et al. (2013) (Supplementary Table 3).

### *A. duranensis* DHN Genes Regulation Under Abiotic and Biotic Stresses

We studied in more detail the regulation of two *A. duranensis* genes (*AdDHN1* and *AdDHN2*) in response to abiotic and biotic stresses. This wild *Arachis* species has been used by our group in functional genomics studies due to its contrasting responses to abiotic and biotic stresses: tolerant to water deficit but somewhat susceptible to *Meloidogyne arenaria* infection (Proite et al., 2008; Leal-Bertioli et al., 2012; Guimaraes et al., 2017). The *in silico* expression profile of *AdDHN1* and *AdDHN2* was determined using our previous RNA-seq data including *A. duranensis* roots submitted to a dehydration treatment (Vinson et al., 2018) and roots challenged with the RKN *M. arenaria* (Mota et al., 2018). We then validated the *in silico* expression analysis based on RNA-seq using qRT-PCR.

The RNA-seq expression profiling of *AdDHN1* transcripts in *A. duranensis* roots showed opposite behaviors for expression of this gene under the two stresses analyzed, with upregulation in response to dehydration and downregulation in response to nematode infection (Supplementary Figure 4). This opposite expression behavior was further validated by qRT-PCR using specific primers (Supplementary Table 2), corroborating the predicted *in silico* analysis (Supplementary Figure 4). Contrastingly, no expression of *AdDHN2* could be detected either *in silico* (no mapped reads) or by qRT-PCR (no amplified samples) under the two stresses evaluated. These results are in accordance with the expression atlas (Supplementary Table 3), which did not show any basal expression of *AdDHN2* in *A. duranensis* roots. Therefore, based on the expression profiling of both *Arachis* DHN genes, only the *AdDHN1* gene was selected for a deeper characterization at functional levels as an interesting candidate involved in opposite responses to abiotic and biotic stresses.

### Analysis of *Arabidopsis* Plants Overexpressing *AdDHN1*

To further investigate the involvement of *AdDHN1* in the response to abiotic and biotic stresses through its overexpression in transgenic plants, a consensus coding sequence was determined. The alignment of five *A. duranensis* sequences showed high nucleotide conservation, except for the gene model Aradu.IF4XP^9^ which present a gap of 128 bp (Supplementary Figure 5). The consensus of *AdDHN1* coding sequence (675 bp) was then cloned and used to produce transgenic *Arabidopsis* plants.

#### Freezing Treatment

**FIGURE 2:**
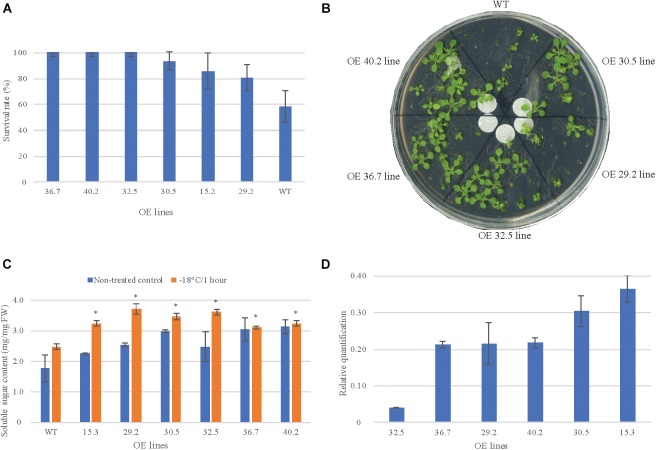
Morphology and freezing tolerance in 2 weeks old *Arabidopsis* wild-type (WT) plants and in six *AdDHN1* overexpressing (OE) lines. **(A)** *Arabidopsis* seedlings and **(B)** their survival rates 3 days after the freezing-shock treatment (-18°C for 1 h); **(C)** Total sugar content in leaves from treated and non-treated control seedlings. Values are means ± SD of three independent replicates and expressed as μg/mg of fresh weight (FW). Significant differences (*P* < 0.05) between WT and OE lines are marked with an asterisk. **(D)** The relative expression of *AdDHN1* transgene quantified by qRT-PCR, using the ACT2 gene from *Arabidopsis* as the reference gene. Error bars are the standard errors of the means from three samples of 10 plants.

The overexpressing (OE) lines at T3 generation were selected based on their response to freezing-shock treatment (survival rate and sugar content) and on the abundance of *AdDHN1* transcripts. We observed an enhanced freezing tolerance in six of the 13 OE lines submitted to freezing treatment (-18°C for 1 h) which showed a greater survival rate than the WT (Figure 2A,B). The plantlets from these six lines (15.2; 29.2; 30.5; 32.5; 36.7; and 40.2) displayed a normal regrowth after the treatment and appeared to be more tolerant to the freezing injury than WT plants, which were not able to resume growth. Moreover, all of these six OE lines accumulated more soluble sugars in leaves than WT plants under normal growth conditions and exhibited a significant increase in sugar content in response to the freezing treatment (Figure 2C). Accumulation of sugars under adverse environmental conditions, including freezing, could contribute to maintain cell turgor and to protect membranes and proteins against stress damage (Krasensky and Jonak, 2012). Accordingly, the overexpression of *AdDHN1* in *Arabidopsis* seemed to improve freezing tolerance concomitant with a higher concentration of solutes, under both normal and stress conditions, as observed in previous studies (Mattana et al., 2005). The qRT-PCR analysis subsequently confirmed the overexpression of the transgene *AdDHN1* in these six OE lines at different expression levels (Figure 2D). Overall, the *AdDHN1* transcript abundance did not correlate with the enhanced freezing tolerance or the accumulation of soluble sugars, in particular for the OE 32.5 line, indicating a possible post-transcriptional regulatory mechanism of the transgene. These findings are in agreement with the general presumption that there is no clear relationship between the transcript levels of overexpressed transgenes and the phenotypic effects observed in transgenic lines, which can vary from a strong positive to no significant correlation (Kerr et al., 2018; Ko et al., 2018). Based on these analyses, the six freezing-tolerant OE lines were selected for further analysis.

#### Dry-Down Assay

**FIGURE 3:**
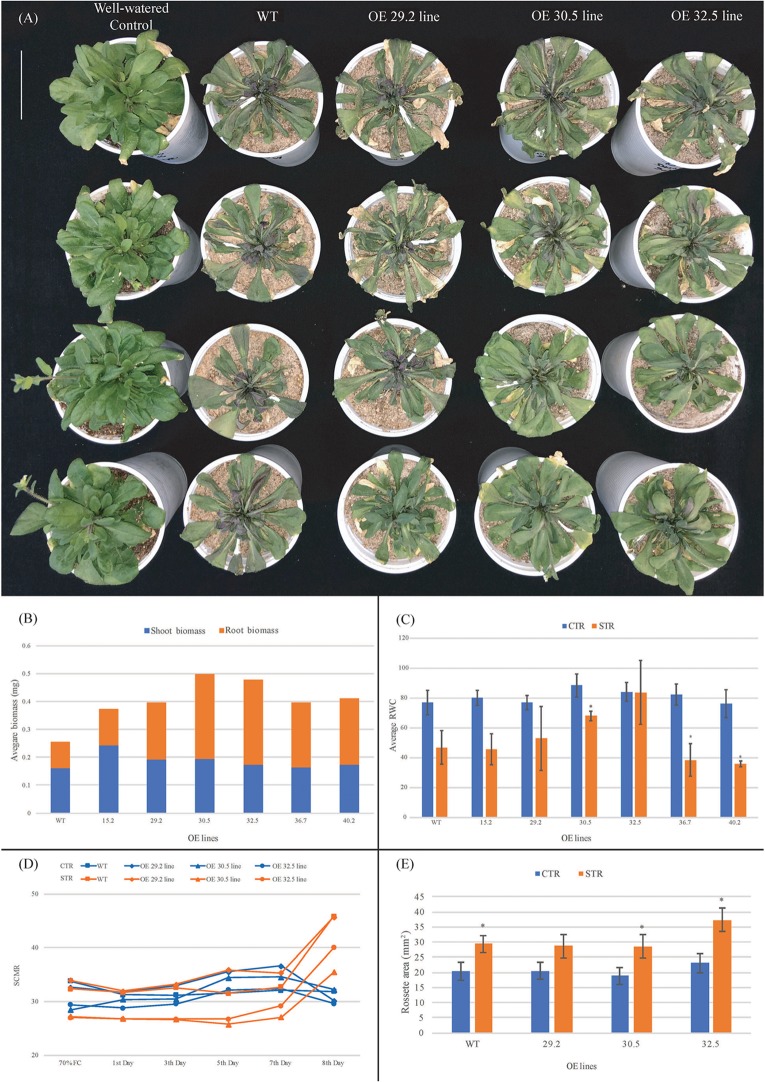
Performance of 3 weeks old *Arabidopsis* plants from WT and *AdDHN1* OE lines submitted to a dry-down treatment for 8 days (stressed plants; STR) and the corresponding irrigated control (control plants; CTR). **(A)** Phenotype of the aerial part of CTR plants (first column) and STR plants (2nd–4th columns) from WT and the three OE lines (29.2, 30.5, and 32.5); **(B)** shoot and root biomass (miligrams of fresh weight) analysis of WT and OE lines; **(C)** Percentage of relative water content (RWC) in leaves from WT and OE lines. Values are means ± SD of 10 individuals and significant (*P* ≤ 0.05) differences between CTR and STR plants are marked with an asterisk; **(D)** SCMR (SPAD chlorophyll meter reads) of WT and OE lines. The dry-down assay initiated with 70% FC, decreasing over time in STR plants from 65% FC (1st Day), 50% FC (3nd Day), 40% FC (5rd Day), 30% FC (7th Day) to 20% FC (8th Day); **(E)** Average of rosette area (mm^2^) of WT and OE plants at 8th day of dry-down treatment. Values are means ± SD of four individuals and significant (*P* ≤ 0.05) differences between CTR and STR plants are marked with an asterisk.

The water deprivation assays indicated that the six best freezing tolerant OE lines also exhibited enhanced drought tolerance, with less morphological damage compared to WT (Figure 3A). From the 5th day of the dry-down assay, WT plants showed progressive symptoms of water deficiency (leaf wilt), followed by growth retardation that resulted in 38.9% lower biomass at the 8th day, compared to the average of OE lines (Figure 3A,B). This lower biomass was particularly noticeable in roots, with around 55.4% less biomass in the WT compared to the average of OEs. The RWC was also analyzed at the end of the dry-down assay, with no significant difference observed between leaf RWC values of OE and WT control plants, with values falling within the expected RWC range of 76–88% (Figure 3C). However, OE and WT stressed plants exhibited differences in the reduction in RWC compared to the corresponding control plants that occurred over the course of the experiment. The reduction of RWC values observed in leaves of three OE lines (29.2; 30.5; and 32.5) in response to water deficit (average of 18%) was significantly smaller than in the other lines (15.2; 36.7; and 40.2) which were similar to the WT plants (around 39%). The OE 32.5 line showed a distinct behavior with a RWC value of 84% under drought conditions, which was almost the same value found for plants under irrigated conditions and a good indicator of drought tolerance due to the capacity of these plants to maintain high leaf water status. These results suggest that leaves of three OE lines (29.2; 30.5; and 32.5) can maintain their levels of internal water higher than WT for eight consecutive days without irrigation. For a more detailed investigation, a new dry-down assay (biological repetition) was carried out with only these three OE lines, under the same experimental conditions. SCMRs recordings confirmed the responsiveness of OE and WT plants to drought imposition that initiated with 70% FC and decreased to 20% FC at the 8th day of treatment (Figure 3D). While the control (irrigated) plants maintained a mean SCMR of 32 over the treatment, stressed (non-irrigated) plants presented a gradual increase from the 3th day, reaching values of approximately 41 five days after. In addition, two OE lines (30.5 and 32.5) displayed reduced SCMR values up to the 6th day of treatment, compared to WT plants, indicating a possible adaptive response to preserve photosynthetic efficiency. Measurements of rosette area showed a significant increase in the total rosette area of WT and OE lines over the course of the stress treatment (Figure 3E). The larger rosette surface area observed in the 32.5 line suggests that this line was better able to maintain growth under stress compared to the WT and in 29.2 and 30.5 OE lines.

#### Nematode Challenge

**FIGURE 4:**
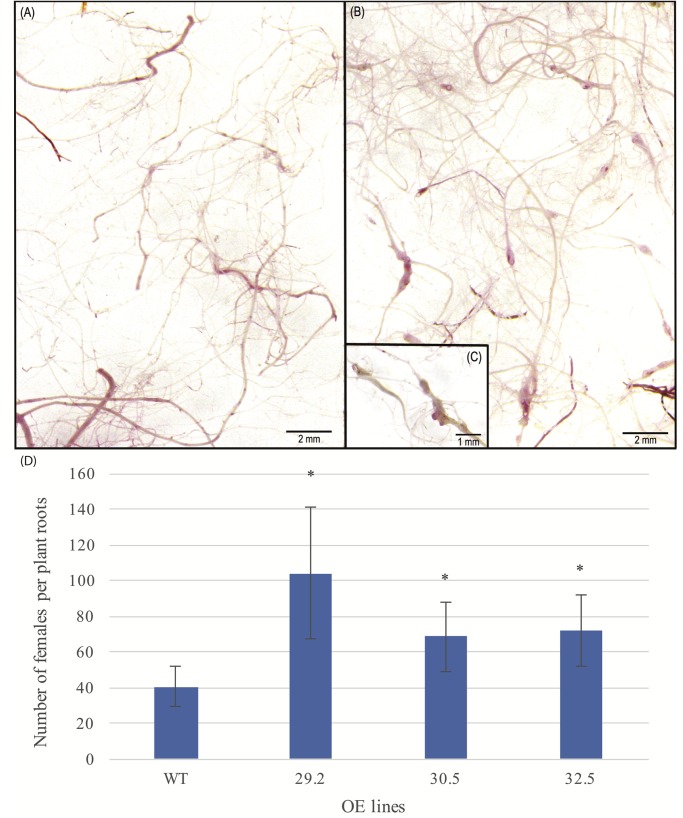
Roots of *Arabidopsis* plants stained by acid fuchsin from **(A)** WT and **(B)** 29.2 OE line, at 60 days after inoculation (DAI) with *Meloidogyne incognita.* **(C)** Zoom in galls observed in 29.2 OE line. **(D)** Average number of females per plant roots of WT and OE lines inoculated with *M. incognita*. Values are means ± SD of 10 individuals and significant (*P* ≤ 0.05) differences between WT and OE lines are marked with an asterisk.

To examine the potential effect of *AdDHN1* overexpression on the nematode infection process in *Arabidopsis*, 4 weeks old OE and WT plants were challenged with 1,000 *M. incognita* juveniles (J2) and the level of infection assessed by the number of nematode females in each root system at 60 DAI. Overall, the three selected OE lines were severely affected by *M. incognita*, with a significant higher number of females found in the roots of all three OE lines, when compared to the WT plants, indicating an increase in the susceptibility due to *AdDHN1* overexpression (Figure 4). The increase in the *M. incognita* infection rates in the OE lines ranged from 60% in 29.2 line to around 41% in 30.5 and 32.5 lines. No visible difference was observed in the morphology and biomass of roots from OE lines and WT, which ranged from 298.5 to 188.8 mg of roots per plant (Figure 4).

#### qRT-PCR Analysis of Stress-Responsive Genes

**FIGURE 5:**
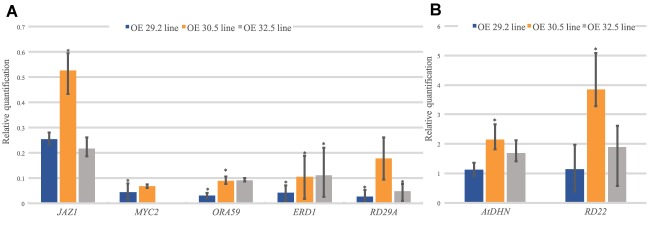
Relative quantification of mRNA levels of seven stress-responsive *Arabidopsis* genes (*JAZ1; MYC2; ORA59; ERD1; RD29A; AtDHN;* and *RD22)* in the three OE lines (29.2, 30.5, and 32.5) relative to the WT plants. **(A)** Downregulated and **(B)** upregulated genes. Values are means ± SD of three biological replicates and significant (*P* ≤ 0.05) differences between WT and OE lines are marked with an asterisk.

**FIGURE 6:**
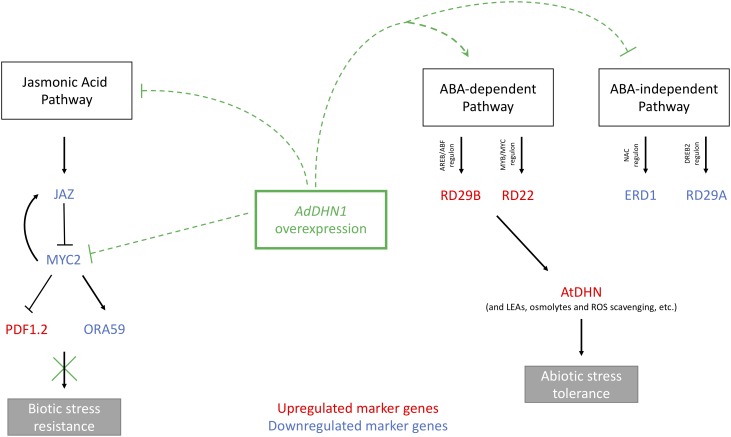
Schematic representation of the ABA and JA pathways in *Arabidopsis*. Black lines represent the expected role of nine marker genes on ABA and JA pathways in WT plants. Green dotted lines represent the hypothetical interference of the *AdDHN1* overexpression in *Arabidopsis* OE lines in these pathways, in accordance with Hanin et al. (2011). The red color indicates marker genes that are upregulated in OE lines and blue color those downregulated.

To determine whether the overexpression of *AdDHN1* could interfere with *Arabidopsis* hormone signaling involved in stress responses, we analyzed the expression of a subset of nine marker genes associated with ABA and JA pathways (Seo et al., 2009; Yang et al., 2011; Sasaki-Sekimoto et al., 2013; Naznin et al., 2014; Guo et al., 2015; Singh and Laxmi, 2015; Zhao et al., 2018; Supplementary Table 2). The relative expression of these marker genes was compared between the three OE lines (29.2, 30.5, and 32.5) and the WT plants. The *AdDHN1* overexpression negatively regulated the expression of two marker genes (*ERD1* and *RD29A*) from the ABA-independent pathway which was both downregulated in the three OE lines (Figure 5A). Conversely, transcripts of the *RD29B* and *RD22* genes from the ABA-dependent pathway seemed to be activated by *AdDHN1* overexpression, as *RD29B* could be detected in two OE lines but not in the WT plants (Supplementary Figure 6) whereas *RD22* was positively regulated in the three OE lines (Figure 5B). Regardless of the apparent interference with ABA-independent signaling, the *AdDHN1* overexpression induced the activation of downstream ABA-responsive genes that promote improvement of drought tolerance, including the orthologous of *AdDHN1* gene in *Arabidopsis* (*AtDHN*) (Figure 5, 6).

Concurrently, *AdDHN1* overexpression promoted the downregulation of three genes involved in the JA pathway (*JAZ1, MYC2*, and *ORA59*; Figure 5A) and the upregulation of *PDF1.2*, a marker gene for JA/ET-mediated responses, which expression was exclusive in OE plants (Supplementary Figure 6). These alterations in the JA pathway could be owed to the blockage of *MYC2* due to *AdDHN1* overexpression, as previously suggested by Hanin et al. (2011) in *Arabidopsis*.

## Discussion

### Fabaceae DHNs Are Mainly of Two Types and Were Already Present in Their Common Ancestor

Dehydrins (DHNs) are LEA proteins known to act in multiple developmental processes and in response to various stresses, and despite their role in abiotic stress tolerance, they remain insufficiently studied in legumes (Fabaceae). The structure in motifs and presence/absence pattern for the whole LEA gene family has previously been described for six legume species (Battaglia et al., 2013). However, this study neither analyzed the evolutionary relationships between the DHN sequences nor their distribution throughout whole genomes in relation to conserved synteny. Here, by using phylogenomics methods and taking advantage of the re-annotated versions of these six genomes, as well as the recent genome sequencing of two *Arachis* species, we provide new insights into molecular evolution of the DHN family in legumes. Based on the presence and conservation of the PFAM domain of DHNs, containing the signature K-segment, we identified 20 likely functional DHN genes in eight species of Fabaceae.

Most DHN (19 out of 20) genes studied here were distributed between two sub-types and all the species, except *C. cajan*, possessed at least one representative of each sub-type, suggesting that the common ancestor of all these species already possessed at least one DHN of each type.

The presence of one type of each DHN in the common ancestor was confirmed by current phylogenetic analysis, which showed a clear separation of DHNs into two distinct groups, those containing the Y-segment (nine Y_n_SK_n_- and one Y_n_K_n_-type), and those without the Y-segment (10 SK_n_-type). However, the McScanX duplication and synteny analysis suggests that the Y_n_SK_n_- and SK_n_-type DHNs in these legumes do not originate from the WGD event described in papilionoid (PWGD) (Cannon et al., 2015). Indeed, the two DHN types tend to be co-localized in close proximity on the same chromosome and were never connected by a WGD/tandem duplication relationship in our analysis. This co-localization on the same chromosome of one Y_n_SK_n_ and one SK_n_-type was probably derived from a common ancestral proximal duplication.

The extra copy of a Y_n_SK_n_-type in *G. max*, in contrast, was clearly due to a WGD event in this species. It is known that *G. max* underwent a WGD that occurred after the differentiation of the other *Glycine* species (Schmutz et al., 2010).

Overall, and despite the PWGD event, the number of DHNs in each Fabaceae species is relatively low when compared to other vascular plant species studied (e.g., six DHN genes per species on average in poplar, *Arabidopsis*, barley and rice (Wang et al., 2007; Bies-Ethève et al., 2008; Hundertmark and Hincha, 2008; Tommasini et al., 2008). This reduced number is likely due to multiple losses of DHN genes over the course of Papilionoideae evolution, through events called Legume Lost Genes (LLGs), recently proposed by Gu et al. (2016).

In Fabaceae, we found full length DHNs for only three of the five known types: nine Y_n_SK_n_ as well as one Y_n_K_n_, both described as more frequent in monocot species (Abedini et al., 2017), and 10 SK_n_-type. Previous studies have described the presence of additional segments, other than the three commonly associated to the DHN genes (K-, S-, and Y-segments), called Φ-segments, preceding the S-segment at the N-terminal region and with no sequence conservation between plant species (Graether and Boddington, 2014; Abedini et al., 2017). These Φ-segments are characterized by an enrichment of glycine and threonine, and, more rarely, tryptophan, cysteine and phenylalanine (Abedini et al., 2017; Malik et al., 2017), however, their function is still not clear. Here, we identified two novel Φ-segments (DRGV[FL]DFLG) and (EE[VA]I[AV]TEF), in the legume DHN genes from the SK_n_ subclass, preceding the S-segment at the N-terminal end of the proteins. These motifs have not yet been described in plant DHNs.

### DHN Segment Organization Does Not Correlate With Their Subcellular Localization but With Their Spatial Expression Pattern

The analyses associating the presence and organization of the conserved segments observed here (K-, S-, and Y-) demonstrated that there is no correlation with the subcellular localization of the DHN genes. However, previously described phosphorylation motifs, may be involved in their location or translocation to the nucleus. The motif LXRXXS can be phosphorylated by a kinase and trigger translocation of the protein from the cytosol to the nucleus. This conserved motif was observed in most of the DHN protein sequences of Fabaceae, explaining the preferential nuclear location of these proteins (Abedini et al., 2017).

While the conserved segments have no influence on DHN subcellular location, it seems to be associated with their expression in different plant tissues. Expression of genes encoding Y_n_SK_n_ proteins was observed preferentially in seeds of the Fabaceae species, agreeing with previous studies in grapevine and *Arabidopsis* (Hundertmark and Hincha, 2008; Yang et al., 2012). Conversely, SK_n_-type DHN genes display a ubiquitous pattern, as they are expressed in both the vegetative tissues (roots, leaves, and stems) and the dry seeds. This difference in the spatial distribution of expression is correlated with the phylogenetic separation of the genes, and could be associated with distinct roles of the subclasses of DHN in the plant, as suggested by Graether and Boddington (2014). The response of the *AdDHN1* gene to drought imposition and its high expression levels in all the analyzed tissues, led to our choosing to clone this gene, and analyze its function further using the model plant *Arabidopsis*.

### Overexpression of *AdDHN1* in Transgenic *Arabidopsis* Plants Led to Better Resistance to Abiotic Stresses but Increased Susceptibility to Nematodes

Several studies have shown the positive effect of DHN overexpression on plant tolerance to different abiotic stress conditions, mainly low temperatures, drought and salinity (Graether and Boddington, 2014). Most of these studies have concentrated on a few DHN genes isolated from model plants or major crops, with few focusing on native species that are well adapted to adverse environmental conditions.

Here, we isolated and studied the DHN gene *AdDHN1* from *A. duranensis*, a wild species native to low rainfall regions in South America that has evolved molecular and morphological adaptation mechanisms that aid survival in adverse and water-limited environments (Leal-Bertioli et al., 2012). We cloned the *AdDHN1* coding sequence under the control of a constitutive promoter to generate transgenic *Arabidopsis* lines expressing this candidate gene, which is potentially involved in both abiotic and biotic stresses. The heterologous overexpression of *AdDHN1* conferred different degrees of tolerance to a freezing-shock treatment in seedlings and to a gradual water deficit assay in substrate-grown plants. These results were supported by plant phenotypes and physiological indices, such as sugar and RWCs, chlorophyll meter readings, biomass and rosette area. This suggests that *AdDHN1* confers a similar protective role in both abiotic stresses and corroborates previous studies showing that the overexpression of SK_n_-type DHNs enhanced the tolerance to low temperatures and drought in transgenic plants (Liu Y. et al., 2015; Bao et al., 2017). Similarly, the overexpression of a YK_n_-type DHN isolated from *Saussurea involucrate*, a wild species that also grows in adverse environmental conditions, also conferred enhanced tolerance to both cold and drought in transgenic tobacco plants (Guo et al., 2017).

Although the role of DHNs in the response to a large range of abiotic stresses is well reported, little is known about their involvement in biotic stress responses. Some studies have reported the induction of DHNs in response to filamentous pathogen attack, such as *Erysiphe necator* in grapevine (Yang et al., 2012) or in combination with water deficit as with *Phytophthora cinnamomi* in oak (Turco et al., 2004), suggesting a putative role of DHNs in modulating pathogen defense responses (Hanin et al., 2011; Rosales et al., 2014). However, to date, the potential effect of DHNs in other interactions with different pathogens, including Metazoa such as nematodes, has not been reported.

Here, in contrast to abiotic stress, the overexpression of *AdDHN1* enhanced the susceptibility of transgenic *Arabidopsis* lines to the RKN *M. incognita*, a biotrophic plant pathogen with a wide host range and a very sophisticated strategy of host colonization. The present study demonstrates for the first time the increased susceptibility to a pathogen attack due to the overexpression of a DHN gene in transgenic plants. However, the mechanisms that are involved in these defense responses, as well as the tradeoffs affecting the plant normal metabolism are still unclear.

### Overexpression of *AdDHN1* in *Arabidopsis* Has Effects on ABA and JA Plant Defense Pathways

It is well known that ABA- and JA-signaling pathways are involved in response to both biotic and abiotic stresses, and, although JA-dependent signaling is generally effective against necrotrophic pathogens (Pieterse et al., 2012), it can also be activated by wounding and some biotrophic pests (Robert-Seilaniantz et al., 2011). However, these hormone signaling pathways often interact in diverse manners in response to a stimulus, a phenomenon referred to as “crosstalk” (Pieterse et al., 2012; Van der Does et al., 2013; Singh and Laxmi, 2015), leading to different, postponed or subdued defense responses.

Many studies support the idea that DHNs are ABA-regulated proteins, as implied by the presence of motifs linked to ABA-dependent and ABA-independent pathways in the promoter of several DHN genes, in particular those containing the S-segment (Zolotarov and Strömvik, 2015; Tiwari et al., 2018; Yu et al., 2018). Here, we showed that the overexpression of *AdDHN1* in transgenic *Arabidopsis* interfered with the expression of a subset of ABA-marker genes in comparison compared to WT plants. For instance, we observed in OE lines a contrasting regulation in the two major ABA signaling pathways, illustrated by the downregulation of *ERD1* and *RD29A*, which are part of the NAC and DREB2 regulons, respectively, in the ABA-independent signal transduction pathway, and the upregulation of *RD22* and *RD29B*, which are part of the MYB/MYC and AREB/ABF regulons, respectively, in the ABA-dependent pathway (Figure 6). These results suggest that the overexpression of *AdDHN1* acts in the ABA-dependent pathway to promote freezing and drought tolerance in transgenic *Arabidopsis* plants, despite the unnoted function of the ABA-independent pathway. This is supported by the induction in OE lines of the endogenous *Arabidopsis* DHN gene (*AtDHN*), which is the orthologous of *AdDHN1*. SK_n_-type DHNs, such as *AtDHN* and *AdDHN1*, are part of the overall macromolecular protection mechanisms activated by the complex transcriptional cascade downstream of the DREB2A stress-regulatory system to avoid water loss during drought stress (Yu et al., 2018). Moreover, Tiwari et al. (2018) recently proposed that, apart from their role as a protective protein, DHNs interact with other proteins to act as a positive regulator involved in ABA-mediated drought stress signaling.

In addition to ABA, other hormone signaling pathways normally associated with biotic and wounding stresses, such as the JA pathway, can also be activated by dehydration stress due to cellular damage (Hanin et al., 2011). In our work, we hypothesized that the overexpression of *AdDHN1* suppresses the expression of *MYC2*, a central regulator in JA synthesis, causing in turn *ORA59* downregulation and enabling the expression of *PDF1.2* (Figure 6). We also found that *JAZ1*, a negative regulator of JA signaling, is also downregulated in the OE lines, possibly through a negative regulatory loop (Chini et al., 2007), due to *MYC2* repression (Figure 6). The overexpression of DHNs causing *MYC2* suppression in transgenic plants and affecting their responses to pathogen attacks has previously been suggested by Hanin et al. (2011). These findings indicate that the interference of *AdDHN1* overexpression in the JA pathway, compromising the modulation of JA- and wound-responsive genes (Hanin et al., 2011), contribute to an increase in nematode susceptibility in the OE lines compared to WT.

Indeed, our recent transcriptome studies of wild *Arachis* showed that both JA and ABA pathways are, similarly, activated in response to dehydration in the drought-tolerant *A. duranensis* (Vinson et al., 2018) whereas the JA pathway is the preferential route triggered in the resistance response to the RKN *M. arenaria* in *A. stenosperma* (Guimaraes et al., 2015; Mota et al., 2018). However, the detailed mechanisms by which the overexpression of *AdDHN1* gene from *A. duranensis* interferes in the responses to dehydration and nematode attack in transgenic *Arabidopsis* and the crosstalk between both stresses remains unclear, although its role in the modulation of ABA and JA signaling pathways are suggested.

Overall, our findings reveal an important tradeoff between biotic and abiotic defense responses following DHN gene overexpression. Although enhanced resistance to abiotic stresses tends to confirm and reinforce the protective role of DHN proteins in abiotic stress tolerance, this comes with a cost for the plant. Indeed, susceptibility to the infection by RKNs is increased, suggesting a negative regulation in disease-resistance responses. Tradeoffs between different processes in plants, including stress responses, may occur due to resource restriction and have an important effect on plant productivity and fitness, with hormone crosstalk having a major role in regulating this balance and prioritizing plant responses. A better understanding of the molecular aspects involved in these defenses signaling interactions is therefore vital to optimize plant engineering programs aiming at improving resistance to abiotic and biotic stresses and their tradeoffs in plants.

## Author Contributions

AM contributed to the design of the work, data analysis and interpretation, and drafting the manuscript. TO and MC performed the data collection and analysis. CV and TW contributed to the data collection and analysis and critical revision of the manuscript. AA and MG-d-S contributed to the data interpretation and critical revision of the manuscript. ED contributed to the design of the work, data analysis and interpretation, and critical revision of the manuscript. PG contributed to the conception and design of the work, data analysis, and critical revision of the manuscript. AB contributed to the conception and design of the work, data analysis, interpretation, drafting, and critical revision of the manuscript. All the authors have approved the final version of the manuscript to be published.

## Conflict of Interest Statement

The authors declare that the research was conducted in the absence of any commercial or financial relationships that could be construed as a potential conflict of interest.

## Footnotes

1 http://omgenomics.com

2 http://gsds.cbi.pku.edu.cn/

3 http://bar.utoronto.ca

4 https://peanutbase.org/

5 http://www.ncbi.nlm.nih.gov

6 http://www.gene-quantification.de/download.html

7 https://wolfpsort.hgc.jp

8 http://bar.utoronto.ca

9 https://peanutbase.org/

## References

[B1] Abedini R. Ghanegolmohammadi F. Pishkamrad R. (2017). Plant dehydrins: shedding light on structure and expression patterns of dehydrin gene family in barley. *J. Plant Res.* 130 747–763. 10.1007/s10265-017-0941-5 28389925

[B2] Araújo S. S. Beebe S. Crespi M. Delbreil B. González E. M. Gruber V. (2015). Abiotic stress responses in legumes: Strategies used to cope with environmental challenges. *Crit. Rev. Plant Sci.* 34 237–280. 10.1080/07352689.2014.898450

[B3] Bailey T. L. Boden M. Buske F. A. Frith M. Grant C. E. Clementi L. (2009). MEME Suite: tools for motif discovery and searching. *Nucleic Acids Res.* 37 202–208. 10.1093/nar/gkp335 19458158 PMC2703892

[B4] Banerjee A. Roychoudhury A. (2016). Group II late embryogenesis abundant (LEA) proteins: structural and functional aspects in plant abiotic stress. *Plant Growth Regul.* 79 1–17. 10.1007/s10725-015-0113-3

[B5] Bao F. Du D. An Y. Yang W. Wang J. Cheng T. (2017). Overexpression of *Prunus mume* dehydrin genes in tobacco enhances tolerance to cold and drought. *Front. Plant Sci.* 8:151. 10.3389/fpls.2017.00151 28224001 PMC5293821

[B6] Battaglia M. Covarrubias A. A. Marina B. Alejandra A. C. (2013). Late embryogenesis abundant (LEA) proteins in legumes. *Front. Plant Sci.* 4:190. 10.3389/fpls.2013.00190 23805145 PMC3691520

[B7] Benedito V. A. Torres-Jerez I. Murray J. D. Andriankaja A. Allen S. Kakar K. (2008). A gene expression atlas of the model legume *Medicago truncatula*. *Plant J.* 55 504–513. 10.1111/j.1365-313X.2008.03519.x 18410479

[B8] Bertioli D. J. Cannon S. B. Froenicke L. Huang G. Farmer A. D. Cannon E. K. S. (2016). The genome sequences of *Arachis duranensis* and *Arachis ipaënsis*, the diploid ancestors of cultivated peanut. *Nat. Genet.* 48 438–446. 10.1038/ng.3517 26901068

[B9] Bies-Ethève N. Gaubier-Comella P. Debures A. Lasserre E. Jobet E. Raynal M. (2008). Inventory, evolution and expression profiling diversity of the LEA (late embryogenesis abundant) protein gene family in *Arabidopsis thaliana*. *Plant Mol. Biol.* 67 107–124. 10.1007/s11103-008-9304-x 18265943

[B10] Buysse J. Merckx R. (1993). An improved colorimetric method to quantify sugar content of plant tissue. *J. Exp. Bot.* 44 1627–1629. 10.1093/jxb/44.10.1627

[B11] Cannon S. B. McKain M. R. Harkess A. Nelson M. N. Dash S. Deyholos M. K. (2015). Multiple polyploidy events in the early radiation of nodulating and nonnodulating legumes. *Mol. Biol. Evol.* 32 193–210. 10.1093/molbev/msu296 25349287 PMC4271530

[B12] Cao Y. Xiang X. Geng M. You Q. Huang X. (2017). Effect of HbDHN1 and HbDHN2 genes on abiotic stress responses in *Arabidopsis*. *Front. Plant Sci.* 8:470. 10.3389/fpls.2017.00470 28443102 PMC5385384

[B13] Capella-Gutiérrez S. Silla-Martínez J. M. Gabaldón T. (2009). trimAl: a tool for automated alignment trimming in large-scale phylogenetic analyses. *Bioinformatics* 25 1972–1973. 10.1093/bioinformatics/btp348 19505945 PMC2712344

[B14] Charfeddine S. Saïdi M. N. Charfeddine M. Gargouri-Bouzid R. (2015). Genome-wide identification and expression profiling of the late embryogenesis abundant genes in potato with emphasis on dehydrins. *Mol. Biol. Rep.* 42 1163–1174. 10.1007/s11033-015-3853-2 25638043

[B15] Chiappetta A. Muto A. Bruno L. Woloszynska M. Lijsebettens M. Van Bitonti M. B. (2015). A dehydrin gene isolated from feral olive enhances drought tolerance in *Arabidopsis* transgenic plants. *Front. Plant Sci.* 6:392. 10.3389/fpls.2015.00392 26175736 PMC4485055

[B16] Chini A. Fonseca S. Fernández G. Adie B. Chico J. M. Lorenzo O. (2007). The JAZ family of repressors is the missing link in jasmonate signalling. *Nature* 448 666–671. 10.1038/nature06006 17637675

[B17] Chu Y. Guimaraes L. A. Wu C. L. Timper P. Holbrook C. C. Ozias-Akins P. (2014). A technique to study Meloidogyne arenaria resistance in *Agrobacterium rhizogenes*-transformed peanut. *Plant Dis.* 98 1292–1299. 10.1094/PDIS-12-13-1241-RE 30703931

[B18] Close T. J. (1996). Dehydrins: Emergence of a biochemical role of a family of plant dehydration proteins. *Physiol. Plant.* 97 795–803. 10.1034/j.1399-3054.1996.970422.x

[B19] Clough S. J. Bent A. F. (1998). Floral dip: a simplified method for *Agrobacterium*-mediated transformation of *Arabidopsis thaliana*. *Plant J.* 16 735–743. 10.1046/j.1365-313x.1998.00343.x 10069079

[B20] de Brito G. G. Sofiatti V. de Andrade Lima M. M. de Carvalho L. P. da Silva Filho J. L. (2011). Physiological traits for drought phenotyping in cotton. *Acta Sci. Agron.* 33 117–125. 10.4025/actasciagron.v33i1.9839

[B21] De Vylder J. Vandenbussche F. Hu Y. Philips W. Van Der Straeten D. (2012). Rosette tracker: an open source image analysis tool for automatic quantification of genotype effects. *Plant Physiol.* 160 1149–1159. 10.1104/pp.112.202762 22942389 PMC3490612

[B22] Finn R. D. Coggill P. Eberhardt R. Y. Eddy S. R. Mistry J. Mitchell A. L. (2016). The Pfam protein families database: towards a more sustainable future. *Nucleic Acids Res.* 44 D279–D285. 10.1093/nar/gkv1344 26673716 PMC4702930

[B23] Graether S. P. Boddington K. F. (2014). Disorder and function: a review of the dehydrin protein family. *Front. Plant Sci.* 5:576. 10.3389/fpls.2014.00576 25400646 PMC4215689

[B24] Grant C. E. Bailey T. L. Noble W. S. (2011). FIMO: scanning for occurrences of a given motif. *Bioinformatics* 27 1017–1018. 10.1093/bioinformatics/btr064 21330290 PMC3065696

[B25] Gu Y. Xing S. He C. (2016). Genome-Wide analysis indicates lineage-specific gene loss during *Papilionoideae* evolution. *Genome Biol. Evol.* 8 635–648. 10.1093/gbe/evw021 26868598 PMC4824202

[B26] Guimaraes L. A. Mota A. P. Z. Araujo A. C. G. de Alencar Figueiredo L. F. Pereira B. M. de Passos Saraiva M. A. (2017). Genome-wide analysis of expansin superfamily in wild *Arachis* discloses a stress-responsive expansin-like B gene. *Plant Mol. Biol.* 94 1–18. 10.1007/s11103-017-0594-8 28243841 PMC5437183

[B27] Guimaraes P. M. Guimaraes L. A. Morgante C. V. Silva O. B. Araujo A. C. G. Martins A. C. Q. (2015). Root transcriptome analysis of wild peanut reveals candidate genes for nematode resistance. *PLoS One* 10:e0140937. 10.1371/journal.pone.0140937 26488731 PMC4619257

[B28] Guo R. Zhao J. Wang X. Wang X. (2015). Constitutive expression of a grape aspartic protease gene in transgenic *Arabidopsis* confers osmotic stress tolerance. *PCTOC* 121 275–287. 10.1007/s11240-014-0699-6

[B29] Guo X. Zhang L. Zhu J. Liu H. Wang A. (2017). Cloning and characterization of SiDHN, a novel dehydrin gene from *Saussurea* involucrata Kar. et Kir. that enhances cold and drought tolerance in tobacco. *Plant Sci.* 256 160–169. 10.1016/j.plantsci.2016.12.007 28167030

[B30] Halder T. Upadhyaya G. Basak C. Das A. Chakraborty C. Ray S. (2018). Dehydrins impart protection against oxidative stress in transgenic tobacco plants. *Front. Plant Sci.* 9:136. 10.3389/fpls.2018.00136 29491874 PMC5817096

[B31] Hanin M. Brini F. Ebel C. Toda Y. Takeda S. Masmoudi K. (2011). Plant dehydrins and stress tolerance: versatile proteins for complex mechanisms. *Plant Signal. Behav.* 6 1503–1509. 10.4161/psb.6.10.17088 21897131 PMC3256378

[B32] Hara M. Fujinaga M. Kuboi T. (2005). Metal binding by citrus dehydrin with histidine-rich domains. *J. Exp. Bot.* 56 2695–2703. 10.1093/jxb/eri262 16131509

[B33] Hill W. Jin X.-L. Zhang X.-H. (2016). Expression of an arctic chickweed dehydrin, CarDHN, enhances tolerance to abiotic stress in tobacco plants. *Plant Growth Regul.* 80 323–334. 10.1007/s10725-016-0169-8

[B34] Houde M. Dallaire S. N’Dong D. Sarhan F. (2004). Overexpression of the acidic dehydrin WCOR410 improves freezing tolerance in transgenic strawberry leaves. *Plant Biotechnol. J.* 2 381–387. 10.1016/j.nicl.2017.06.031 17168885

[B35] Hundertmark M. Hincha D. K. (2008). LEA (late embryogenesis abundant) proteins and their encoding genes in *Arabidopsis thaliana*. *BMC Genomics* 9:118. 10.1186/1471-2164-9-118 18318901 PMC2292704

[B36] Jiménez-Bremont J. F. Maruri-López I. Ochoa-Alfaro A. E. Delgado-Sánchez P. Bravo J. Rodríguez-Kessler M. (2013). lea gene introns: is the intron of dehydrin genes a characteristic of the serine-segment? *Plant Mol. Biol. Rep.* 31 128–140. 10.1007/s11105-012-0483-x

[B37] Katoh K. Misawa K. Kuma K. Miyata T. (2002). MAFFT: a novel method for rapid multiple sequence alignment based on fast fourier transform. *Nucleic Acids Res.* 30 3059–3066. 10.1093/nar/gkf436 12136088 PMC135756

[B38] Kerr T. C. C. Abdel-mageed H. Aleman L. Lee J. Payton P. Cryer D. (2018). Ectopic expression of two AREB / ABF orthologs increases drought tolerance in cotton (*Gossypium hirsutum*). *Plant Cell Environ.* 41 898–907. 10.1111/pce.12906 28098349

[B39] Ko D. K. Nadakuduti S. S. Douches D. S. Buell C. R. (2018). Transcriptome profiling of transgenic potato plants provides insights into variability caused by plant transformation. *PLoS One* 13:e0206055. 10.1371/journal.pone.0206055 30408049 PMC6224046

[B40] Kovacs D. Kalmar E. Torok Z. Tompa P. (2008). Chaperone activity of ERD10 and ERD14, two disordered stress-related plant proteins. *Plant Physiol.* 147 381–390. 10.1104/pp.108.118208 18359842 PMC2330285

[B41] Krasensky J. Jonak C. (2012). Drought, salt, and temperature stress-induced metabolic rearrangements and regulatory networks. *J. Exp. Bot.* 63 1593–1608. 10.1093/jxb/err460 22291134 PMC4359903

[B42] Leal-Bertioli S. C. M. Bertioli D. J. Guimaraes P. M. Pereira T. D. Galhardo I. Silva J. P. (2012). The effect of tetraploidization of wild *Arachis* on leaf morphology and other drought-related traits. *Environ. Exp. Bot.* 84 17–24. 10.1016/j.envexpbot.2012.04.005

[B43] Liang Y. Xiong Z. Zheng J. Xu D. Zhu Z. Xiang J. (2016). Genome-wide identification, structural analysis and new insights into late embryogenesis abundant (LEA) gene family formation pattern in *Brassica napus*. *Sci. Rep.* 6:24265. 10.1038/srep24265 27072743 PMC4829847

[B44] Liu H. Yu C. Li H. Ouyang B. Wang T. Zhang J. (2015). Overexpression of ShDHN, a dehydrin gene from *Solanum habrochaites* enhances tolerance to multiple abiotic stresses in tomato. *Plant Sci.* 231 198–211. 10.1016/j.plantsci.2014.12.006 25576005

[B45] Liu Y. Zhang J. Li W. Guo C. Shu Y. (2015). In silico identification, phylogeny and expression analysis of expansin superfamily in *Medicago truncatula*. *Biotechnol. Biotechnol. Equip.* 30 197–203. 10.1080/13102818.2015.1093919

[B46] Malik A. A. Veltri M. Boddington K. F. Singh K. K. Graether S. P. (2017). Genome analysis of conserved dehydrin motifs in vascular plants. *Front. Plant Sci.* 8:709. 10.3389/fpls.2017.00709 28523013 PMC5415607

[B47] Mattana M. Biazzi E. Consonni R. Locatelli F. Vannini C. Provera S. (2005). Overexpression of Osmyb4 enhances compatible solute accumulation and increases stress tolerance of *Arabidopsis thaliana*. *Physiol. Plant* 125 212–223. 10.1111/j.1399-3054.2005.00551.x

[B48] Mistry J. Finn R. D. Eddy S. R. Bateman A. Punta M. (2013). Challenges in homology search: HMMER3 and convergent evolution of coiled-coil regions. *Nucleic Acids Res.* 41:e121. 10.1093/nar/gkt263 23598997 PMC3695513

[B49] Morgante C. V. Brasileiro A. C. M. Roberts P. A. Guimaraes L. A. Araujo A. C. G. Fonseca L. N. (2013). A survey of genes involved in arachis stenosperma resistance to meloidogyne arenaria race 1. *Funct. Plant Biol.* 40 1298–1309. 10.1071/FP13096 32481196

[B50] Morgante C. V. Guimaraes P. M. Martins A. Araujo A. C. G. Leal-Bertioli S. C. M. Bertioli D. J. (2011). Reference genes for quantitative reverse transcription-polymerase chain reaction expression studies in wild and cultivated peanut. *BMC Res. Notes* 4:339. 10.1186/1756-0500-4-339 21906295 PMC3180468

[B51] Mota A. P. Z. Vidigal B. Danchin E. G. J. Togawa R. C. Leal-Bertioli S. C. M. Bertioli D. J. (2018). Comparative root transcriptome of wild Arachis reveals NBS-LRR genes related to nematode resistance. *BMC Plant Biol.* 18:159. 10.1186/s12870-018-1373-7 30081841 PMC6080386

[B52] Naznin H. A. Kiyohara D. Kimura M. Miyazawa M. Shimizu M. Hyakumachi M. (2014). Systemic resistance induced by volatile organic compounds emitted by plant growth-promoting fungi in *Arabidopsis thaliana*. *PLoS One* 9:e86882. 10.1371/journal.pone.0086882 24475190 PMC3903595

[B53] Pfaffl M. W. Horgan G. W. Dempfle L. (2002). Relative expression software tool (REST) for group-wise comparison and statistical analysis of relative expression results in real-time PCR. *Nucleic Acids Res.* 30:36. 10.1093/nar/30.9.e36 11972351 PMC113859

[B54] Pieterse C. M. J. Van der Does D. Zamioudis C. Leon-Reyes A. Van Wees S. C. M. (2012). Hormonal modulation of plant immunity. *Annu. Rev. Cell Dev. Biol.* 28 489–521. 10.1146/annurev-cellbio-092910-154055 22559264

[B55] Proite K. Carneiro R. Falcão R. Gomes A. Leal-Bertioli S. Guimarães P. (2008). Post-infection development and histopathology of *Meloidogyne arenaria* race 1 on *Arachis* spp. *Plant Pathol.* 57 974–980. 10.1111/j.1365-3059.2008.01861.x

[B56] Rémus-Borel W. Castonguay Y. Cloutier J. Michaud R. Bertrand A. Desgagnés R. (2010). Dehydrin variants associated with superior freezing tolerance in alfalfa (*Medicago sativa* L.). *Theor. Appl. Genet.* 120 1163–1174. 10.1007/s00122-009-1243-7 20039014

[B57] Robert-Seilaniantz A. Grant M. Jones J. D. G. (2011). Hormone crosstalk in plant disease and defense: more than just jasmonate-salicylate antagonism. *Annu. Rev. Phytopathol.* 49 317–343. 10.1146/annurev-phyto-073009-114447 21663438

[B58] Rosales R. Romero I. Escribano M. I. Merodio C. Sanchez-Ballesta M. T. (2014). The crucial role of Φ- And K-segments in the in vitro functionality of *Vitis vinifera* dehydrin DHN1a. *Phytochemistry* 108 17–25. 10.1016/j.phytochem.2014.10.006 25457499

[B59] Sasaki-Sekimoto Y. Jikumaru Y. Obayashi T. Saito H. Masuda S. Kamiya Y. (2013). Basic helix-loop-helix transcription factors JASMONATE-ASSOCIATED MYC2-LIKE1 (JAM1), JAM2, and JAM3 are negative regulators of jasmonate responses in *Arabidopsis*. *Plant Physiol.* 163 291–304. 10.1104/pp.113.220129 23852442 PMC3762649

[B60] Schmutz J. Cannon S. B. Schlueter J. Ma J. Mitros T. Nelson W. (2010). Genome sequence of the palaeopolyploid soybean. *Nature* 463 178–183. 10.1038/nature08670 20075913

[B61] Seo P. J. Xiang F. Qiao M. Park J. Lee Y. N. Kim S. (2009). The MYB96 transcription factor mediates abscisic acid signaling during drought stress response. *Plant Physiol.* 151 275–289. 10.1104/pp.109.144220 19625633 PMC2735973

[B62] Shekhawat U. K. S. Srinivas L. Ganapathi T. R. (2011). MusaDHN-1, a novel multiple stress-inducible SK3-type dehydrin gene, contributes affirmatively to drought- and salt-stress tolerance in banana. *Planta* 234 915–932. 10.1007/s00425-011-1455-3 21671068

[B63] Shen Y. Tang M. J. Hu Y. L. Lin Z. P. (2004). Isolation and characterization of a dehydrin-like gene from drought-tolerant *Boea crassifolia*. *Plant Sci.* 166 1167–1175. 10.1016/j.plantsci.2003.12.025

[B64] Singh D. Laxmi A. (2015). Transcriptional regulation of drought response: a tortuous network of transcriptional factors. *Front. Plant Sci.* 6:895. 10.3389/fpls.2015.00895 26579147 PMC4625044

[B65] Stamatakis A. (2006). RAxML-VI-HPC: maximum likelihood-based phylogenetic analyses with thousands of taxa and mixed models. *Bioinformatics* 22 2688–2690. 10.1093/bioinformatics/btl446 16928733

[B66] Tiwari P. Indoliya Y. Singh P. K. Singh P. C. Chauhan P. S. Pande V. (2018). Role of dehydrin-FK506-binding proteins complex in enhancing drought tolerance through ABA-mediated signaling pathway. *Environ. Exp. Bot.* 158 136–149. 10.1016/j.envexpbot.2018.10.031

[B67] Tommasini L. Svensson J. T. Rodriguez E. M. Wahid A. Malatrasi M. Kato K. (2008). Dehydrin gene expression provides an indicator of low temperature and drought stress: transcriptome-based analysis of Barley (*Hordeum vulgare* L.). *Funct. Integr. Genomics* 8 387–405. 10.1007/s10142-008-0081-z 18512091

[B68] Turco E. Close T. J. Fenton R. D. Ragazzi A. (2004). Synthesis of dehydrin-like proteins in *Quercus ilex* L. and *Quercus cerris* L. seedlings subjected to water stress and infection with *Phytophthora cinnamomi*. *Physiol. Mol. Plant Pathol.* 65 137–144. 10.1016/j.pmpp.2004.11.010

[B69] Van der Does D. Leon-Reyes A. Koornneef A. Van Verk M. C. Rodenburg N. Pauwels L. (2013). Salicylic acid suppresses jasmonic acid signaling downstream of SCFCOI1-JAZ by targeting GCC promoter motifs via transcription factor ORA59. *Plant Cell* 25 744–761. 10.1105/tpc.112.108548 23435661 PMC3608790

[B70] Vinson C. C. Mota A. P. Z. Oliveira T. N. Guimaraes L. A. Leal-Bertioli S. C. M. Williams T. C. R. (2018). Early responses to dehydration in contrasting wild Arachis. *PLoS One* 13:e0198191. 10.1371/journal.pone.0198191 29847587 PMC5976199

[B71] Wang H. Zhang H. Li Z. (2007). Analysis of gene expression profile induced by water stress in upland rice (*Oryza sativa* L. var. IRAT109) seedlings using subtractive expressed sequence tags library. *J. Integr. Plant Biol.* 49 1455–1463. 10.1111/j.1672-9072.2007.00553.x

[B72] Wang Y. Tang H. DeBarry J. D. Tan X. Li J. Wang X. (2012). MCScanX: a toolkit for detection and evolutionary analysis of gene synteny and collinearity. *Nucleic Acids Res.* 40:e49. 10.1093/nar/gkr1293 22217600 PMC3326336

[B73] Xie C. Zhang R. Qu Y. Miao Z. Zhang Y. Shen X. (2012). Overexpression of MtCAS31 enhances drought tolerance in transgenic *Arabidopsis* by reducing stomatal density. *New Phytol.* 195 124–135. 10.1111/j.1469-8137.2012.04136.x 22510066

[B74] Yamasaki Y. Koehler G. Blacklock B. J. Randall S. K. (2013). Dehydrin expression in soybean. *Plant Physiol. Biochem.* 70 213–220. 10.1016/j.plaphy.2013.05.013 23792826

[B75] Yang S. Seo P. J. Yoon H. Park C. (2011). The *Arabidopsis* NAC transcription factor VNI2 integrates abscisic acid signals into leaf senescence via the COR / RD genes. *Plant Cell* 23 2155–2168. 10.1105/tpc.111.084913 21673078 PMC3160032

[B76] Yang Y. He M. Zhu Z. Li S. Xu Y. Zhang C. (2012). Identification of the dehydrin gene family from grapevine species and analysis of their responsiveness to various forms of abiotic and biotic stress. *BMC Plant Biol.* 12:140. 10.1186/1471-2229-12-140 22882870 PMC3460772

[B77] Yin Z. Rorat T. Szabala B. M. Ziółkowska A. Malepszy S. (2006). Expression of a *Solanum sogarandinum* SK3-type dehydrin enhances cold tolerance in transgenic cucumber seedlings. *Plant Sci.* 170 1164–1172. 10.1016/j.plantsci.2006.02.002

[B78] Yu Z. Wang X. Zhang L. (2018). Structural and functional dynamics of dehydrins: A plant protector protein under abiotic stress. *Int. J. Mol. Sci.* 19:3420. 10.3390/ijms19113420 30384475 PMC6275027

[B79] Zhang H. Zheng J. Su H. Xia K. Jian S. Zhang M. (2018). Molecular cloning and functional characterization of the dehydrin (IpDHN) gene from Ipomoea pes-caprae. *Front. Plant Sci.* 9:1454. 10.3389/fpls.2018.01454 30364314 PMC6193111

[B80] Zhao J. Zhang X. Guo R. Wang Y. Guo C. Li Z. (2018). Over-expression of a grape WRKY transcription factor gene, VlWRKY48, in *Arabidopsis thaliana* increases disease resistance and drought stress tolerance1. *Plant Cell Tissue Organ Cult.* 132 359–370. 10.1007/s11240-017-1335-z

[B81] Zhao S. Fernald R. D. (2005). Comprehensive algorithm for quantitative real-time polymerase chain reaction. *J. Comput. Biol.* 12 1047–1064. 10.1089/cmb.2005.12.1047.Comprehensive 16241897 PMC2716216

[B82] Zolotarov Y. Strömvik M. (2015). De novo regulatory motif discovery identifies significant motifs in promoters of five classes of plant dehydrin genes. *PLoS One* 10:e0129016. 10.1371/journal.pone.0129016 26114291 PMC4482647

