# Supplementary material for: Contrasting Effects of Wild Arachis Dehydrin Under Abiotic and Biotic Stresses
Source: Front Plant Sci. 2019 Apr 18;10:497. doi: 10.3389/fpls.2019.00497 (PMC6482428; doi:10.3389/fpls.2019.00497)
Supplement: Supplementary file 1 [file Data_Sheet_1.pdf]

## *Supplementary Material*

# Contrasting effects of wild *Arachis* dehydrin under abiotic and biotic stresses

Ana Paula Zotta Mota[1,2], Thais Nicolini Oliveira[1,3], Christina Cleo Vinson[1,3], Thomas Christopher Rhys Williams[3], Marcos Mota do Carmo Costa[1], Ana Claudia Guerra Araujo[1], Etienne G.J. Danchin[4], Maria Fatima Grossi-de-Sá[1], Patricia Messenberg Guimaraes[1], Ana Cristina Miranda Brasileiro[1]

*** Corresponding author:** ana.brasileiro@embrapa.br

**Supplementary Tables and Figures**

**Supplementary Table 1:** Putative DHN genes in eight Fabaceae species: Gene annotation, DHN subclass classification, presence of Φ-segments and phosphorylation motifs and protein size and cellular location.

| *Fabaceae species Gene ID* | Gene annotation | DHN subclass | Φ-segment | Phosphorylation motifs (LXRXXS) | Nuclear localization site (RRKK) | Protein length (aa) | Molecular weight (kDa) | Cellular location |
|---|---|---|---|---|---|---|---|---|
| ***Arachis duranensis*** [a] | | | | | | | | |
| Aradu.IF4XP | AdDHN1 | $SK_2$ | Yes | Yes | No | 192 | 21.12 | Nuclear |
| Aradu.L7CNH | AdDHN2 | $Y_2SK_2$ | No | No | Yes | 207 | 22.77 | Nuclear |
| ***Arachis ipaënsis*** [a] | | | | | | | | |
| Araip.TR541 | AiDHN1 | $Y_2SK_2$ | No | No | Yes | 208 | 22.88 | Nuclear |
| Araip.3269G | AiDHN2 | $SK_2$ | Yes | Yes | No | 179 | 19.69 | Nuclear |
| ***Cicer arietinum*** [b] | | | | | | | | |
| Ca_14461 | CaDHN1 | $Y_2SK_2$ | No | Yes | Yes | 184 | 22 | Cytoplasmic |
| Ca_14457 | CaDHN2 | $SK_2$ | Yes | Yes | No | 200 | 20.24 | Nuclear |
| ***Cajanus cajan*** [c] | | | | | | | | |
| C.cajan_11145 | CcDHN1 | $SK_2$ | Yes | Yes | No | 203 | 22.33 | Nuclear |
| C.cajan_18977 | CcDHN2 | $SK_3$ | Yes | Yes | No | 216 | 23.76 | Nuclear |
| ***Glycine max*** [d] | | | | | | | | |
| Glyma.04G009400.1 | GmDHN1 | $SK_3$ | Yes | Yes | No | 214 | 23.54 | Nuclear |
| Glyma.04G009900.1 | GmDHN2 | $Y_2SK_2$ | No | Yes | Yes | 166 | 18.26 | Nuclear |
| Glyma.12G235800.1 | GmDHN3 | $Y_2SK$ | No | No | No | 135 | 14.85 | Nuclear |
| Glyma.13G201300.1 | GmDHN4 | $Y_2SK$ | No | No | No | 139 | 15.29 | Nuclear |
| ***Lotus japonicus*** [e] | | | | | | | | |
| Chr1.CM0113.680 | LjDHN1 | $SK_2$ | Yes | Yes | No | 207 | 22.77 | Nuclear |
| Chr1.CM0113.750 | LjDHN2 | $Y_2SK_2$ | No | Yes | Yes | 153 | 16.83 | Nuclear |
| Chr2.CM0310.270 | LjDHN3 | $YK_4$ | No | No | No | 315 | 34.65 | Nuclear |
| Chr5.CM0335.70 | LjDHN4 | $SK_3$ | Yes | No | No | 233 | 25.63 | Nuclear |
| ***Medicago truncatula*** [d] | | | | | | | | |
| Medtr3g117190.1 | MtDHN1 | $YSK_2$ | No | Yes | Yes | 196 | 21.56 | Nuclear |
| Medtr3g117290.1 | MtDHN2 | $SK_2$ | Yes | Yes | No | 209 | 22.99 | Nuclear |
| ***Phaseolus vulgaris*** [d] | | | | | | | | |
| Phvul.009G004400.1 | PvDHN1 | $SK_2$ | Yes | Yes | No | 202 | 22.2 | Nuclear |
| Phvul.009G005300.1 | PvDHN2 | $Y_2SK_2$ | No | Yes | Yes | 177 | 19.47 | Nuclear |

[a] http://peanutbase.org
[b] https://legumeinfo.org
[c] http://gigadb.org/dataset/100028
[d] https://phytozome.jgi.doe.gov
[e] https://lotus.au.dk/data/download

**Supplementary Table 2:** Primers used for qRT-PCR analysis.

| Primer name | Primer sequence (5´ - 3´) Forward/Reverse | Reference |
|---|---|---|
| eGFP | GAGCTGAAGGGCATCGACTT / TTCTGCTTGTCGGCCATGAT | Endo et al. (2014) |
| pPZP | CTACCAGAATTTGGCTTGAC / GTAAAACGACGGCCAGT | Pereira et al. (2018) |
| AdDHN1 | TTGTCGTCAAACTCGGTGGCGA / TCATGGCAGAGGAGCACCACAA | Vinson et al. (2018) |
| AdDHN2 | GGTGCAACCAGAGAGAAAGG / ACCTTGTCCGTCATCCTCAG | This study |
| AtACT2 | CTTGCACCAAGCAGCATGAA / CCGATCCAGACACTGTACTTCCTT | Czechowski et al. (2005) |
| AtEF-1α | TGAGCACGCTCTTCTTGCTTTCA / GGTGGTGGCATCCATCTTGTTACA | Czechowski et al., (2005) |
| AtPDF1.2 | GAAGCACAGAAGTTGTGCGA / TGTAACAACAACGGGAAAATAAACA | Zhao et al., (2018) |
| AtDREB2A | AAGGGTCGAAGAAGGGTTGT / CGAGCCAAAGGACCATACAT | This study |
| AtRD29B | AAAAGAGAGGCACCGACTCA / CCGTTGACCACCGAGATAGT | This study |
| AtORA59 | CCCCGGAGAACTCTTCTTCA / CACGGTGGCTTCTTTTCCTT | Sasaki-Sekimoto et al., (2013) |
| AtJAZ1 | GAGCAAAGGCACCGCTAATA / TGCGATAGTAGCGATGTTGC | Sasaki-Sekimoto et al., (2013) |
| AtMYC2 | AGCAACGTTTACAAGCTTTGATTG / TCATACGACGGTTGCCAGAA | Naznin et al., (2014) |
| AtDHN | AAGGCTGTGGGATGTTTGAC / AGGCTTCTCCTCTTCCTTGG | This study |
| AtRD22 | CGTTTCAACGTCTCCGAAAA / GACCCAATAGAGTAGCCGGG | Seo et al., (2009) |
| AtRD29A | GTTACTGATCCCACCAAAGAAGA / GGAGACTCATCAGTCACTTCCA | Yang et al., (2011) |
| AtERD1 | GTCAAGATGAGGCGGTAGC / GTCCACAGAAAAGCATAGCAG | Guo et al., (2015) |

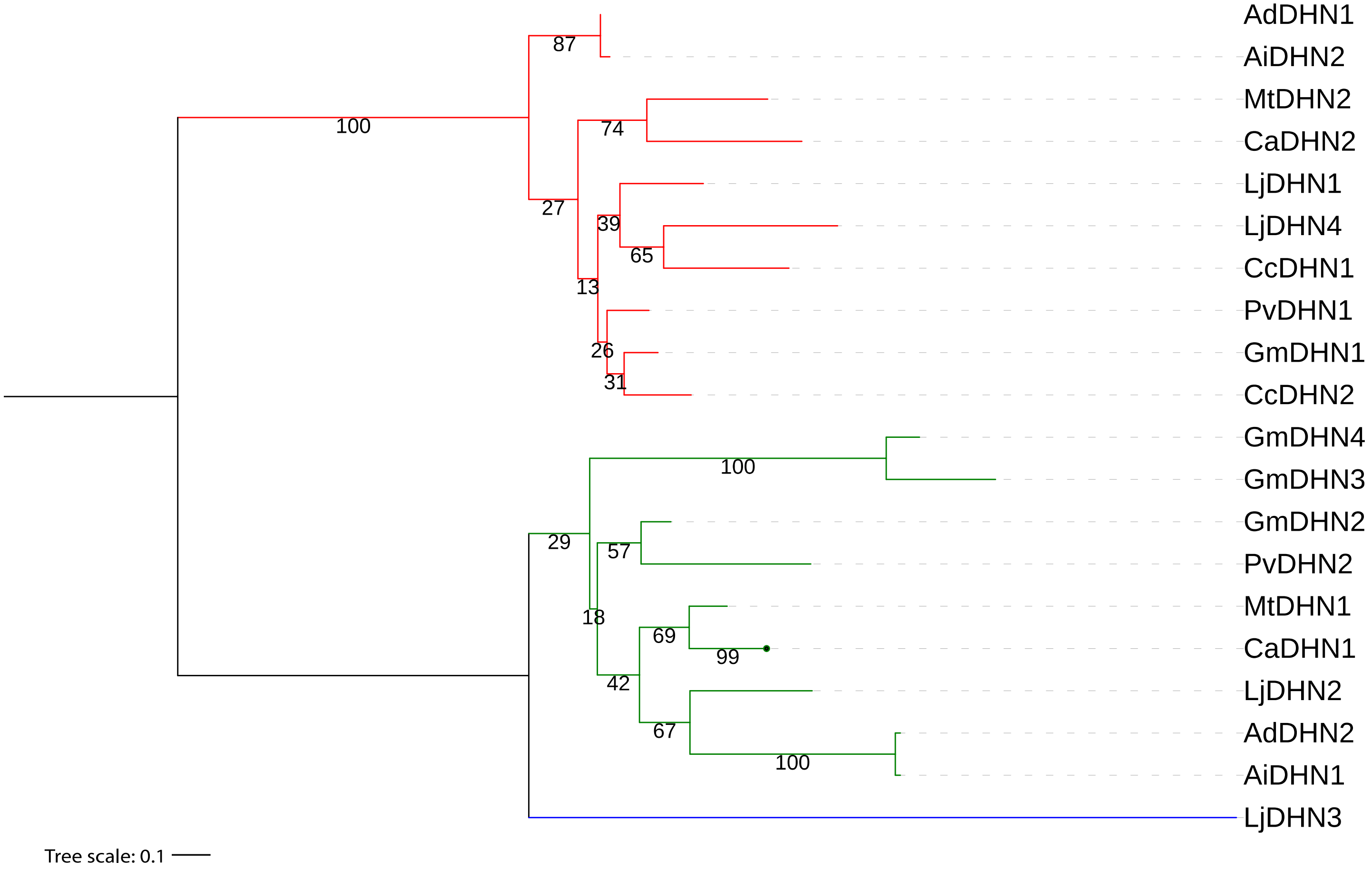


**Supplementary Figure 1:** Phylogenetic tree of the 20 deduced DHN protein sequences identified in eight Fabaceae species: *Arachis duranensis* (Ad), *Arachis ipaënsis* (Ai), *Cicer arietinum* (Ca), *Cajanus cajan* (Cc), *Glycine max* (Gm), *Lotus japonicus* (Lj), *Medicago truncatula* (Mt), and *Phaseolus vulgaris* (Pv). Subclasses $SK_n$ (red), $Y_nSK_n$ (green) and $Y_nK_n$ (blue).

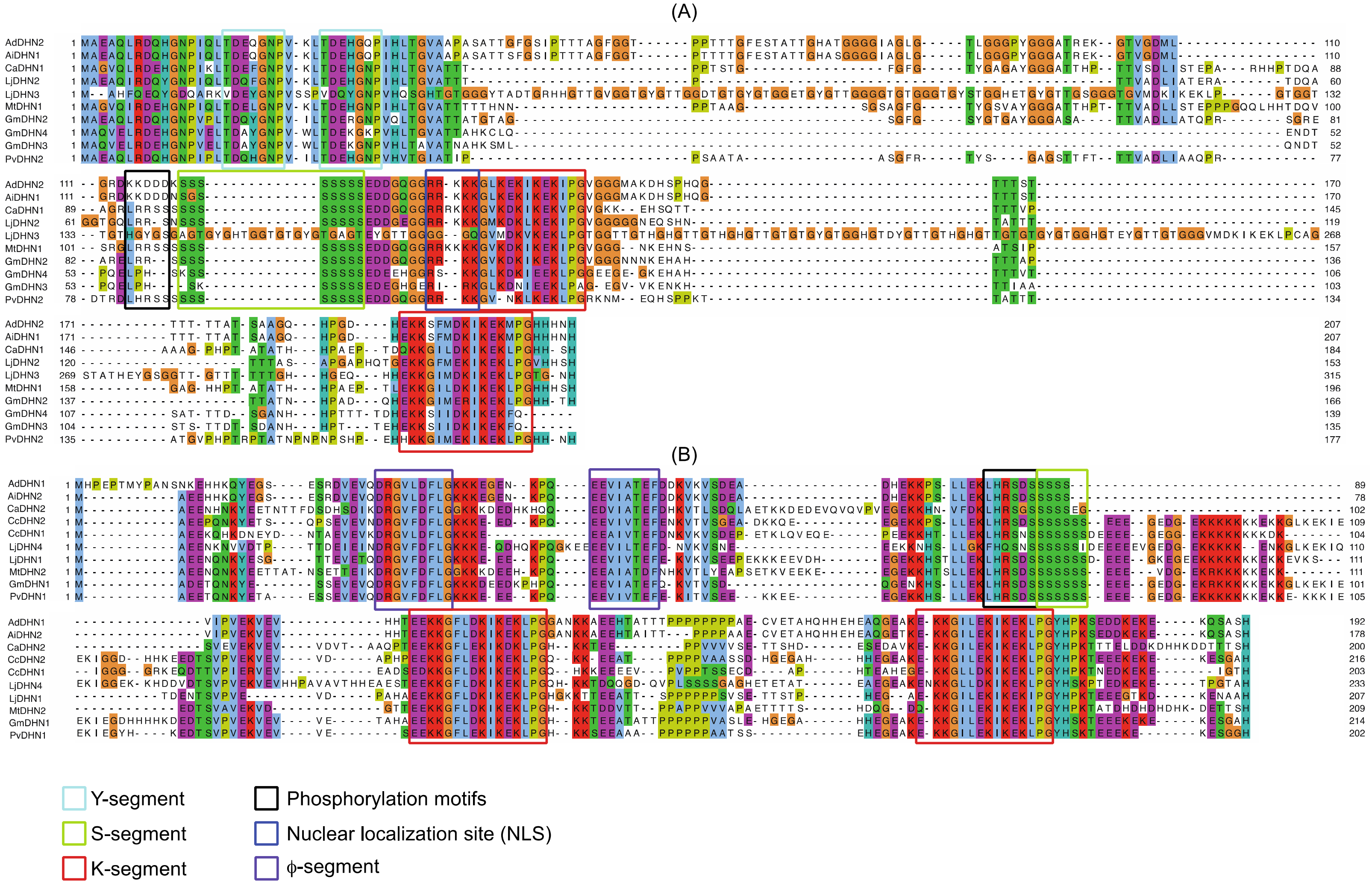


**Supplementary Figure 2:** Alignment of the 20 DHN proteins from eight Fabaceae species. (A) Proteins belonging to the $Y_nSK_n$ subclass. (B) Proteins belonging to the $SK_n$ subclass. The conserved Y-, S-, K-segments, phosphorylation and ϕ-segment are represented by blue, green, red, black and purple squares, respectively.

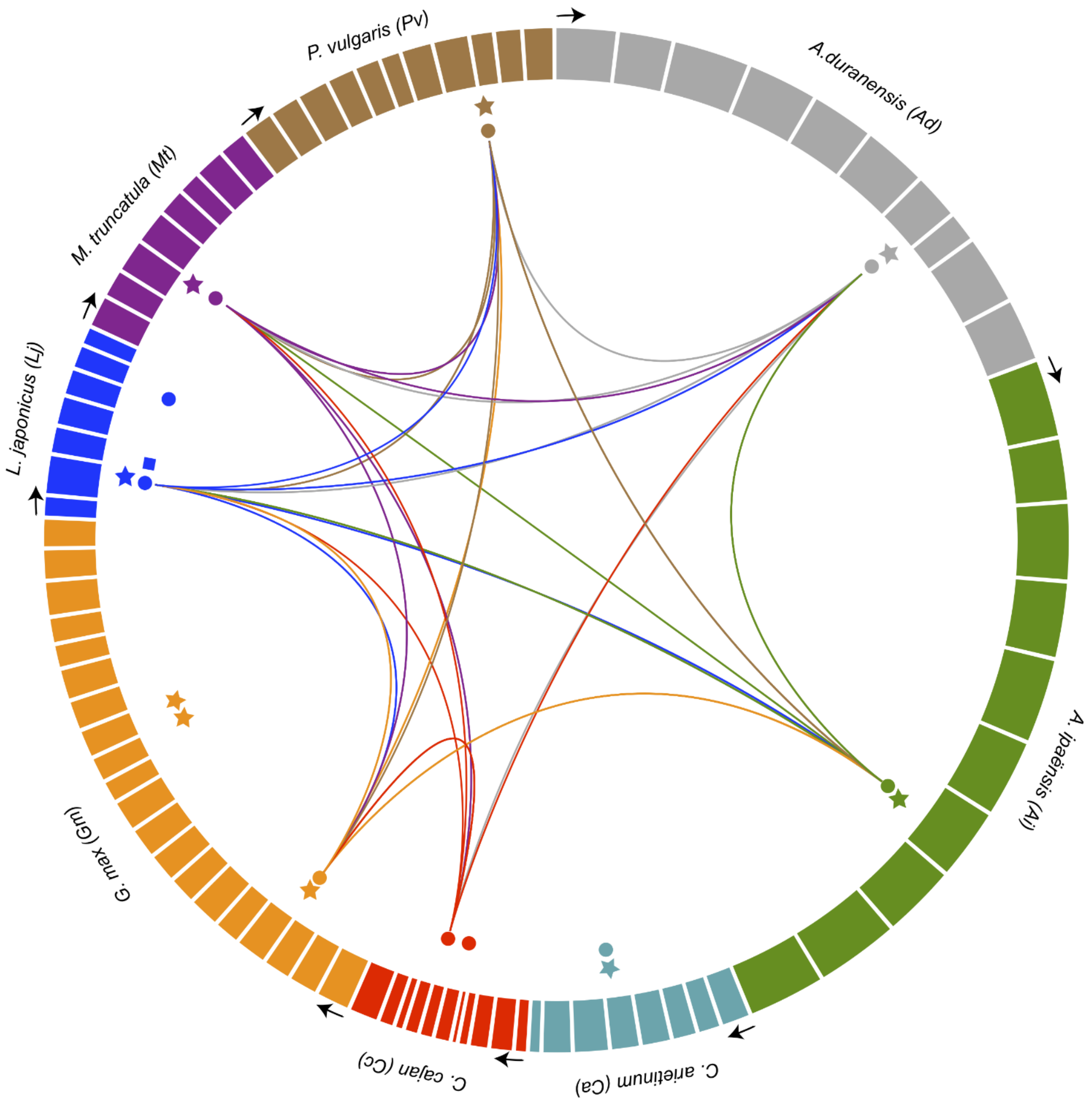


**Supplementary Figure 3:** Chromosomal location and syntenic relationships of the 20 DHN genes in eight species of Fabaceae. Block colors represent the different species and the arrows indicates the first chromosome of each species. Circles represent $SK_n$-type genes, stars $Y_nSK_n$-type and squares $Y_nK_n$-type. The syntenic relationships between the genes are represented by colored lines.

**Supplementary Table 3**: Expression atlas values (average of the FPKM) of DHN genes in four tissues from *Arachis duranensis* and *Arachis ipaënsis*, *Glycine max* and *Medicago truncatula* (http://bar.utoront.ca).

| DHN subclass | $SK_n$-type DHN | | | | $Y_nSK_n$-type DHN | | | | | |
|---|---|---|---|---|---|---|---|---|---|---|
| Tissue | ***AdDHN1*** | ***AiDHN2*** | ***GmDHN1*** | ***MtDHN2*** | ***AdDHN2*** | ***AiDHN1*** | ***GmDHN2*** | ***GmDHN3*** | ***GmDHN4*** | ***MtDHN1*** |
| Dry seeds | 36.81 | 58.53 | n/a* | n/d | 560.46 | 312.61 | n/a* | n/a* | n/a* | 16397.275 |
| Roots | 71.95 | 99.98 | 449.51 | n/d | 0 | 0 | n/d | 0 | 0 | 65.29 |
| Leaves | 16.31 | 22.295 | 356.18* | n/d | 0.04 | 0 | n/d | 0 | 0 | 116.05 |
| Stems | 4.26 | 6.67 | 216.57* | n/d | 0 | 0 | n/d | 0 | 0 | 214.5 |

n/d: Not detected. Transcripts not detected in any of the analysed tissues (http://bar.utoront.ca).

n/a: Not Applicable. Seeds were not analysed in *G. max* expression atlas.

* Gene expression detected by RT-PCR analysis in seeds, leaves and stems of *G. max* (Yamasaki et al, 2013).

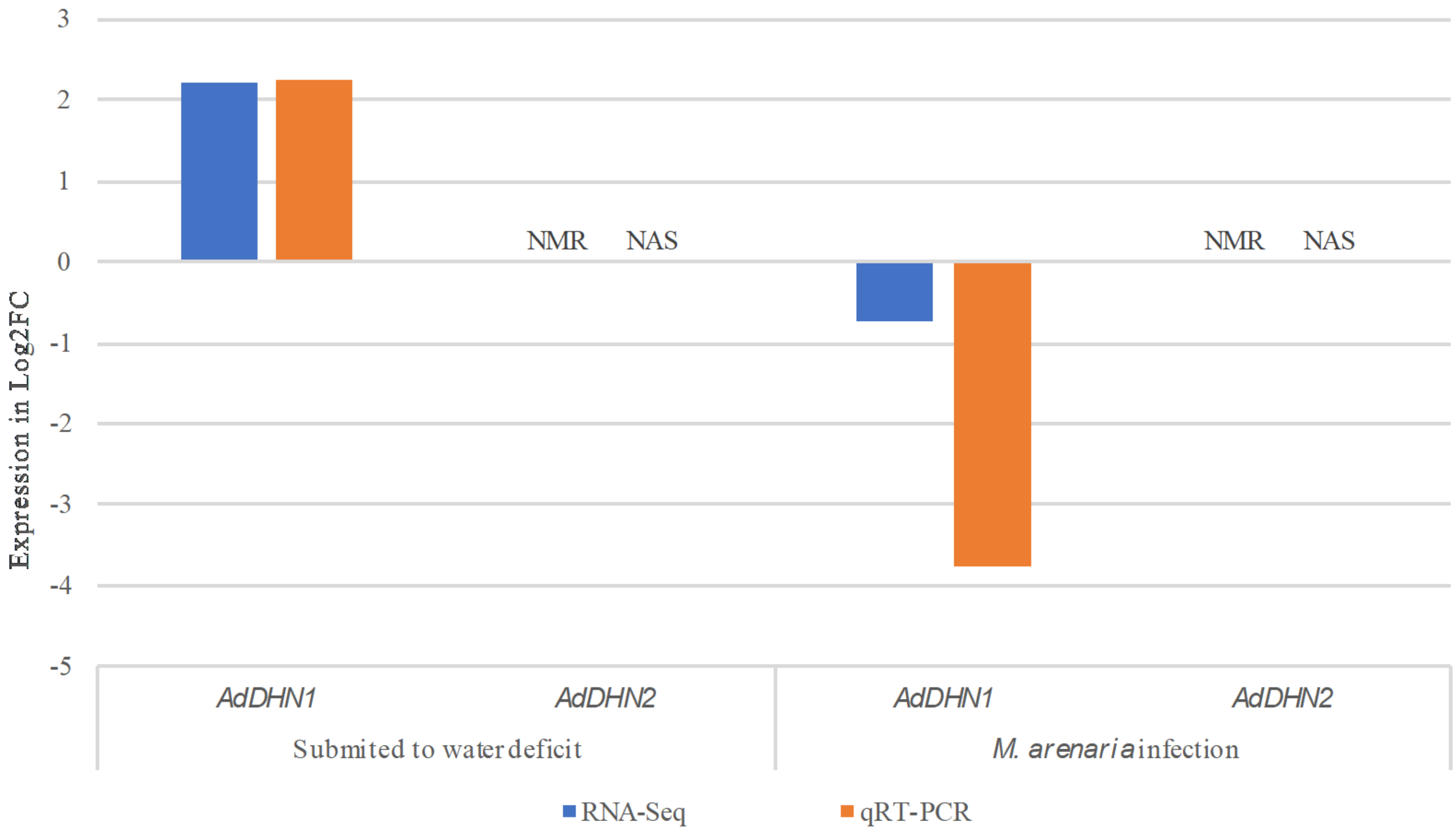


**Supplementary Figure 4:** Expression of AdDHN1 and AdDHN2 submitted to drought and nematode infection. The values of the RNA-Seq data are represented in Log2FC and the relative expression for the RT-qPCR. (NMR) Not mapped samples. (NAS) Not amplified samples.

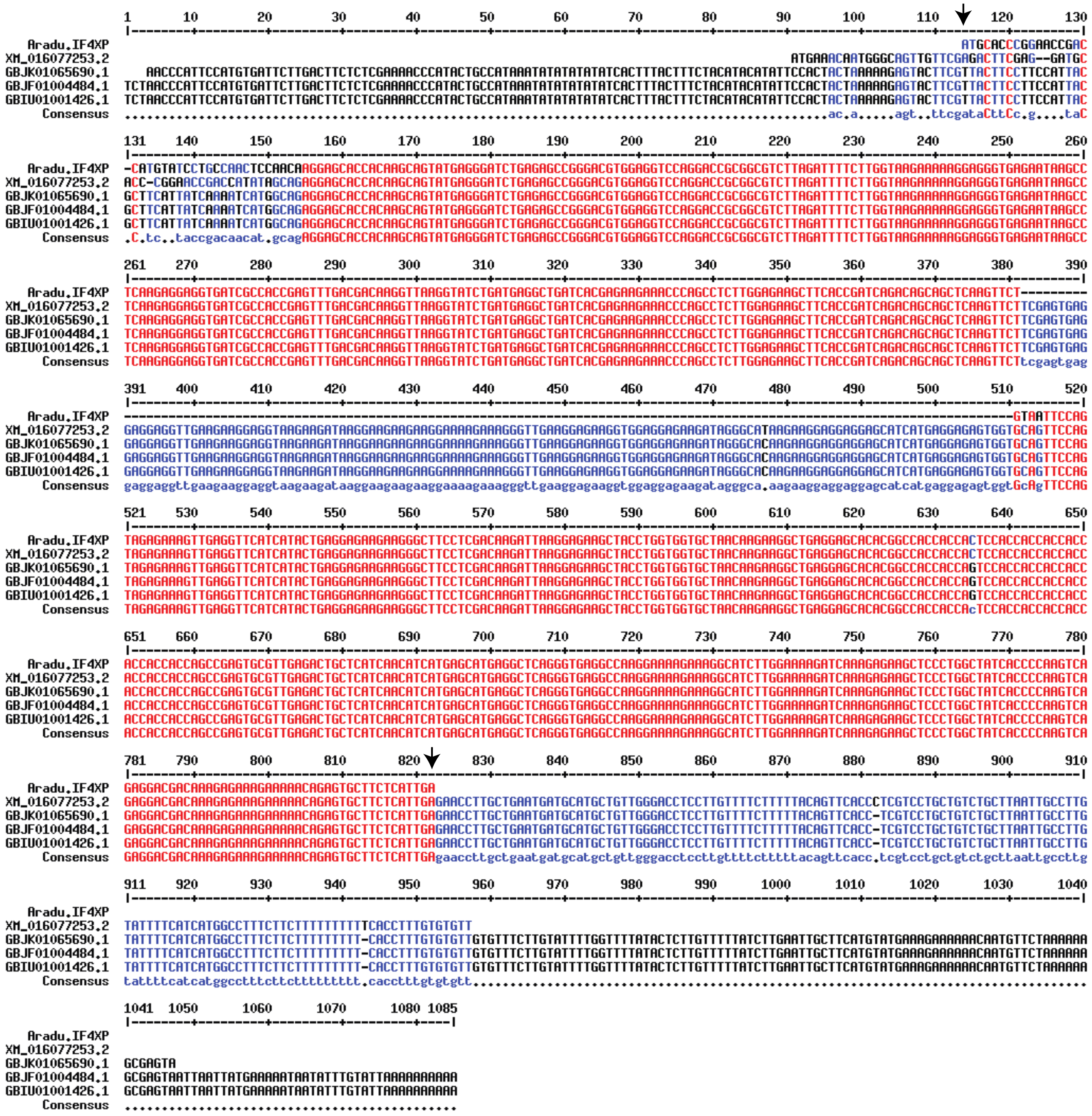


**Supplementary Figure 5:** Alignment of five sequences to determine the consensus sequence of *AdDHN1*. The sequences XM_016077253.2, GBJK01065690.1, GBJF01004484.1 and GBIU01001426.1 were retrieved from NCBI database of TSA of *Arachis duranensis*, and Aradu.IF4XP was retrieved from http://peanutbase.com from the *Arachis duranensis* database.

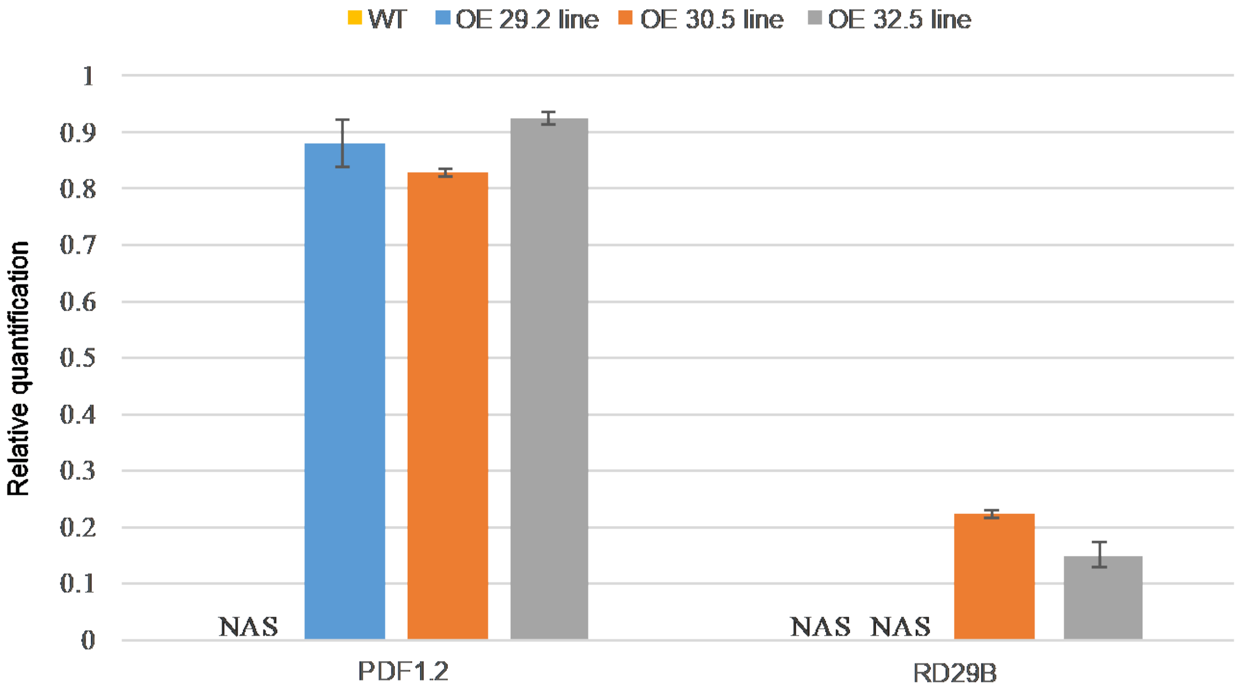


**Supplementary Figure 6:** Relative quantification of two stress-responsive genes (*PFD1.2 and RD29B)* from *Arabidopsis* in the three OE lines (29.2, 30.5 and 32.5) and the WT. Expression profiles of the two genes were determined by qRT-PCR, using the EF1 gene from *Arabidopsis* as the reference gene. Error bars are the standard errors of the means from three samples of ten plants.
